# Polyamine metabolism impacts T cell dysfunction in the oral mucosa of people living with HIV

**DOI:** 10.1038/s41467-023-36163-2

**Published:** 2023-01-25

**Authors:** S. S. Mahalingam, S. Jayaraman, N. Bhaskaran, E. Schneider, F. Faddoul, A. Paes da Silva, M. M. Lederman, R. Asaad, K. Adkins-Travis, L. P. Shriver, P. Pandiyan

**Affiliations:** 1 Department of Biological Sciences, School of Dental Medicine, Case Western Reserve University, Cleveland, OH 44106 USA; 2 Advanced Education in General Dentistry, School of Dental Medicine, Case Western Reserve University, Cleveland, OH 44106 USA; 3 Department of Periodontics, School of Dental Medicine, Case Western Reserve University, Cleveland, OH 44106 USA; 4 Department of Medicine, Division of Infectious Diseases & HIV Medicine, Case Western Reserve University, Cleveland, OH 44106 USA; 5 University Hospitals Cleveland Medical Center AIDS Clinical Trials Unit, Cleveland, OH 44106 USA; 6 Department of Chemistry, Center for Metabolomics and Isotope Tracing, Washington University, Saint Louis, MO 63110 USA; 7 Department of Pathology, School of Medicine, Case Western Reserve University, Cleveland, OH 44106 USA; 8 Center for AIDS Research, School of Medicine, Case Western Reserve University, Cleveland, OH 44106 USA; 9 Present Address: Faculty of Biomedical Sciences, Sri Ramachandra Institute of Higher Education and Research, Chennai, India

**Keywords:** Antimicrobial responses, Mechanisms of disease, Retrovirus

## Abstract

Metabolic changes in immune cells contribute to both physiological and pathophysiological outcomes of immune reactions. Here, by comparing protein expression, transcriptome, and salivary metabolome profiles of uninfected and HIV+ individuals, we found perturbations of polyamine metabolism in the oral mucosa of HIV+ patients. Mechanistic studies using an in vitro human tonsil organoid infection model revealed that HIV infection of T cells also resulted in increased polyamine synthesis, which was dependent on the activities of caspase-1, IL-1β, and ornithine decarboxylase-1. HIV-1 also led to a heightened expression of polyamine synthesis intermediates including ornithine decarboxylase-1 as well as an elevated dysfunctional regulatory T cell (T_regDys_)/T helper 17 (Th17) cell ratios. Blockade of caspase-1 and polyamine synthesis intermediates reversed the T_regDys_ phenotype showing the direct role of polyamine pathway in altering T cell functions during HIV-1 infection. Lastly, oral mucosal T_regDys_/Th17 ratios and CD4 hyperactivation positively correlated with salivary putrescine levels, which were found to be elevated in the saliva of HIV+ patients. Thus, by revealing the role of aberrantly increased polyamine synthesis during HIV infection, our study unveils a mechanism by which chronic viral infections could drive distinct T cell effector programs and T_reg_ dysfunction.

## Abstract (web summary)

Polyamine metabolism is a determinant of T helper cell polarization. Here, Mahalingam *et al* analyse the metabolic and transcriptomic profile of oral mucosa from people living with HIV to demonstrate the effect of polyamine synthesis on T cell dysfunction during HIV-1 infection.

## Introduction

How viruses alter the metabolic states of immune cells and the precise mechanisms underlying the persisting immune dysfunction during chronic viral infections are key questions that have not been fully addressed. Given that discrete tissue microenvironments govern diversity, function, and plasticity of immune cells that in turn regulate anti-viral responses and inflammatory signals, devising effective and targeted approaches require a comprehensive understanding of tissue-specific immunology. In the era of combined anti-retroviral therapy (cART), HIV infection is characterized by systemic inflammation, persisting mucosal immune cell dysfunction, and increased risk of biological aging and associated comorbidities. People living with HIV (PLWH) represent ~37.7 million [30.2–45.1 million] of the world population with a significant compromise on the quality of life and no current treatments for co-morbidities in PLWH^1^. Higher prevalence of periodontitis, oral candidiasis, and cancers are observed in PLWH, although the underlying mechanisms are less studied^2,3^. Oral mucosal T helper (Th) dysregulation and hyperactivation coincide with enhanced toll like receptor (TLR) and NLR family pyrin domain containing (NLRP)3-driven inflammasome signaling, elevated levels of FOXP3^+^T_regs_ and their dysfunction in PLWH^4^. Our previous study has shown that HIV infection associated Th dysregulation involved an enrichment of dysfunctional FOXP3^+^ cells (T_regDys_) defined by PD-1^hi^IFN-γ^+^FOXP3^+^CD4^+^ markers. Although higher expression of PD-1 and IL-1β contributed to T_regDys_ induction and proliferation, the precise mechanism of overall Th dysregulation in PLWH is unclear. Polyamine synthesis represents one of the fundamental processes and has recently been shown to determine the functional fates of Th subsets^5–7^. Ornithine decarboxylase (ODC-1) is the rate-limiting enzyme in polyamine biosynthesis pathway and converts L-ornithine to the first polyamine putrescine, which is sequentially converted into spermidine and spermine^8^. Spermidine acts as a substrate in the process of hypusination of eukaryotic translation initiation factor 5A (EIF5A), which is upregulated during T cell receptor (TCR) activation and regulates cytokine expression in Th cells^5^. EIF5A hypusination is a post-translational modification of a specific lysine residue that requires two consecutive enzymatic steps involving deoxyhypusine synthase (DHPS), and deoxyhypusine hydroxylase (DOHH). These components of polyamine synthesis pathway were recently implicated in altering Th activation, cytokine expression, and cellular fidelity^5^.

Here we show that HIV-mediated alterations in polyamine metabolism contribute to the mechanism of tilting the balance between Th17 and T_reg_ cell subsets in the oral mucosa of PLWH. Our previous study characterized the T_regDys_ subset during HIV infection and showed that T_regDys_ induction and expansion requires caspase-1 activity and IL-1β release^4^. Here we found that pyroptotic loss of Th17 cells coupled with the skewing of the Th balance towards T_regDys_ phenotype also requires ODC-1 mediated polyamine synthesis and EIF5A hypusination, which are also enhanced during HIV infection. ODC-1 and polyamines do not directly regulate Th17 cell frequencies in the context of HIV infection. However, blockade of ODC-1 activity as well as EIF5A hypusination using N1-guanyl-1,7-diaminoheptane (GC7), a spermidine analog reduced the frequency of T_regDys_ during HIV infection in vitro. The addition of exogenous polyamines also induced amphiregulin (AREG) upregulation and proliferation of T_regDys_. Importantly in PLWH, increased T_reg_/Th17 ratio and CD4 hyperactivation positively correlated with the upregulation of polyamine putrescine in the oral mucosa. In conclusion, our study uncovers the role of polyamine metabolism as a cardinal determinant of Th dysregulation and chronic inflammation in the context of viral diseases.

## Results

### RNA-sequencing of oral gingival mucosa and salivary metabolome analysis show dysregulation of arginine and polyamine pathways in PLWH

**Fig. 1:**
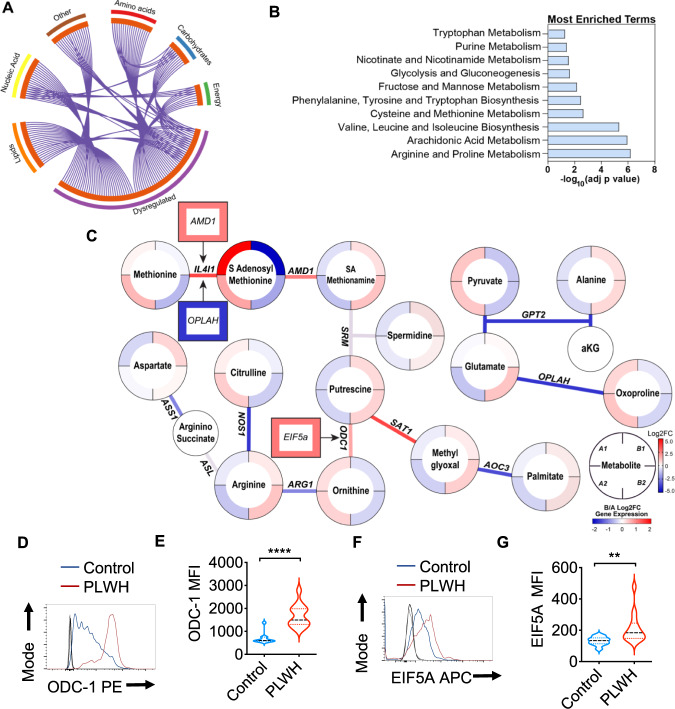
Salivary metabolome analysis in conjunction with transcriptome and flow cytometry analysis of gingival immune cells. Metabolite levels were determined by LC-MS in saliva samples from healthy controls (Group A) and PLWH (Group B) (uninfected controls *n* = 26; PLWH; *n* = 40). Genes are mapped to pathways (Kegg, GO, Reactome), and the algorithm calculates the fold change and *p*-value between how many hits were input and how many hits would be expected from random chance. Log10 adjp-value indicates the significance of each term compared to random chance, larger values indicate higher likelihood that the pathway is truly enriched. Pathways are analyzed individually with no comparisons made. **A** Circos plot linking genes significantly dysregulated between groups A and B (purple outer ring) to their associated KEGG pathways via purple line. Graph was generated via Metascape. **B** Overall the most enriched terms by -log10p value were generated using dysregulated genes and metabolites via Metaboanalyst 5.0. **C** Genes and metabolites associated with the most enriched term, Arginine proline metabolism. Circles represent metabolites ringed by Log2FC (PLWH vs Controls). Lines represent enzymatic genes colored by Log2FC. Rectangles indicate genes not directly involved in enzymatic reactions but are upstream or downstream of the linked metabolites, ringed by Log2FC. Metabolite changes were considered significant if they reached a threshold of Log2FC = 1, *p* < 0.05. Cells from gingival mucosa were processed for flow cytometry and stained for intracellular ODC-1 (Control *n* = 10; PLWH n = 13) (**D**, **E**) and EIF5A (Control *n* = 10; PLWH *n* = 14) (**F**, **G**). Light and dark gray histograms represent staining controls for Control and PLWH cells respectively. Flow cytometry analysis was conducted gating on CD4^+^ T cells. **E** and **G** show the respective statistical analysis of geometric mean fluorescence intensity (MFI). Median values ± SEM are plotted. (Mann–Whitney U test ****P < 0.0001; **P = 0.007; Two-tailed). Circles within box plots represent each replicate.

Using global metabolomic profiling of saliva combined with transcriptional profiling of the oral gingival mucosa, we first identified metabolic alterations in cART treated PLWH versus uninfected controls. We combined the transcriptomic and metabolomics datasets using a combined computational approach that annotates gene expression changes associated with alterations in metabolite pool sizes. Linked gene/metabolite associations were subsequently mapped to pathways using MetaboAnalyst and Metascape. Metabolite identifications were made with Compound Discoverer 3.1 SP1 and MZmine2 software. MS1 and MS2 spectra were matched using a mass tolerance of m/z = 0.1. The raw data were acquired and aligned using the Compound Discover based on the m/z value and the retention time of ion signals. Ions from both ESI− or ESI+ were merged and imported into the SIMCA-P program (version 14.1) for multivariate analysis. Further data processing was performed by using DecoID (DecoID v0.3.0) to deconvolute chimeric MS2 spectra and increase the identification rate. Metabolite identifications were made with level 2 confidence according to the Metabolomics Standards Initiative^9^. Metabolite changes were considered significant if they reached a threshold of Log2FC = 1, *p* < 0.05. Using these metabolite and gene lists, we examined pathways significantly altered by HIV infection (Group B; *n* = 40) compared to uninfected controls (Group A; *n* = 26). The enrichment analysis indicated a widespread metabolic reprogramming within the oral cavity associated with HIV infection. Partial Least-Squares Discriminant Analysis (PLS-DA) and Variable Importance in Projection (VIP) score plots are shown in Supplementary Fig. 1. Alterations in metabolite classes included amino acids, energy metabolites, lipids, and carbohydrates. Together, these changes were reflected in the top enriched pathway hits that include amino acid utilization (arginine and proline, tryptophan, and branched-chain amino acids), arachidonic acid metabolism, and the use of carbohydrates (Fig. 1A, B). Activated immune cells have previously been shown to upregulate glucose utilization to support proliferation, differentiation, and effector functions such as cytokine secretion^10^. Therefore, we examined the changes in expression of glycolytic genes in PLWH versus controls. Enzymes that control glycolytic flux including phosphofructokinase (PFKFB3) are strongly upregulated by HIV infection (Log2FC 2.68, *p* = 1.15 × 10^4^) (Supplementary Fig. 2). Several transcriptional regulators of hypoxic and glycolytic responses and responses to glucose deprivation such as hypoxia-inducible factor 1 (HIF-1)α and DNA damage-inducible transcript 4 protein (DDIT4) were also enriched in PLWH in gingival mucosa. Interestingly, glucose was also increased in PLWH while the immediate downstream intermediates glucose 6-phosphate and fructose 6-phosphate were decreased compared to healthy controls (Supplementary Fig. 3). However, no significant changes in glycolytic and TCA metabolites, or lactate were noted in PLWH (Supplementary Fig. 3). Arachidonic acid is also a key lipid mediator driving inflammatory responses^11,12^. Our transcriptomic and metabolomic datasets were significantly enriched for hits in this pathway (Supplementary Fig. 2). Therefore, we examined enzymes and metabolites involved in the production of eicosanoids and leukotrienes within the oral mucosa. Arachidonic acid is significantly increased (*p* = 0.049) in saliva along with the eicosinoids, 5-hydroperoxyeicosatetraenoic acid (5-HPETE), and 12-hydroxyeicosatetraenoic acid (12-HETE)^13^. Increased levels of 5-HPETE and 12-HETE were also consistent with the upregulation of their biosynthetic enzymes arachidonate 5-lipoxygenase (ALOX5) and CYP4F3 detected in the transcriptomic analysis. Moreover, we detected perturbations in the tryptophan/ kynurenine axis in PLWH. This pathway consumes tryptophan to produce molecules such as kynurenine that regulate immune cell function^14^. Kynurenine also functions as a ligand for aryl hydrocarbon receptors, the activation of which promotes T_reg_ differentiation^15^. Although heightened tryptophan catabolism and kynurenine levels would be consistent with the T_reg_ enrichment that we showed previously in PLWH^4^, we found an opposite trend, including upregulation of kynureninase, moderately lower levels of kynurenine, and increased levels of tryptophan in saliva in oral mucosa of PLWH (Supplementary Fig. 2).

Based on the pathway enrichment analysis data, we further examined other pathways that may be related to immune activation in the oral mucosa. We subsequently examined gene/metabolite relationships driving the alterations in amino acid metabolism by mapping the dysregulated genes to associated metabolites (Log2FC cutoff =1) using metabolomics computational tools, including MetaboAnalyst and DecoID^16^. From this analysis, we identified key nodes altered by HIV infection that are involved in the production and use of amino acids including arginine (Fig. 1C). To determine age-dependent changes in control (Group A) and PLWH (Group B), we stratified the groups based on their age (A1 and B1 aged <60 and A2 and B2 age >60). HIV infected individuals showed significant downregulation of several enzymes involved in glutamate utilization or production, including 5-oxoprolinase (OPLAH, Log2FC = −1.64, *p* = 0.016), and alanine aminotransferase (GPT2, Log2 FC = −1.64, *p* = 0.007) independent of age-stratification (Fig. 1C). Reduced expression of GPT2 impacts the production of alanine from pyruvate and is consistent with alterations in the activity of this enzyme, alanine levels were significantly increased, while pyruvate is decreased in the saliva of PLWH. Interestingly, individuals with HIV showed increases in arginine in saliva while the pool size of citrulline was decreased compared to healthy controls (Fig. 1C). This led us to examine nitric oxide and ornithine pathways. Several enzymes including two enzymes that play a role in polyamine synthesis; spermidine/spermine N(1)-acetyltransferase (SAT1) and ornithine decarboxylase (ODC-1) show increased transcription in PLWH. The precursor of polyamines, ornithine is also decreased in PLWH, while ODC-1, the polyamine putrescine and *EIF5A* transcript downstream to putrescine were elevated relative to healthy controls indicating that there may be increased polyamine synthesis in the microenvironment of the oral mucosa (Fig. 1C). Because ODC-1 was recently shown to instruct Th subset fates^5,17^, we hypothesized that HIV-induced altered metabolic states could impact T_reg_/Th dysregulation in oral mucosa of PLWH^4^. Consistent with the upregulation of *ODC-1* and *EIF5A* transcripts in PLWH (Fig. 1C), flow cytometry analyses also revealed heightened expression of these proteins in CD4^+^ T cells of PLWH individuals when compared to uninfected controls (Fig. 1D–G; gating strategy; Supplementary Fig. 4). Altogether, the oral mucosa of HIV infected patients provided an important insight into how polyamine metabolism may be relevant to altering T cell functions in mucosae.

### HIV-1 infection causes T_reg_/Th17 ratio skewing

**Fig. 2:**
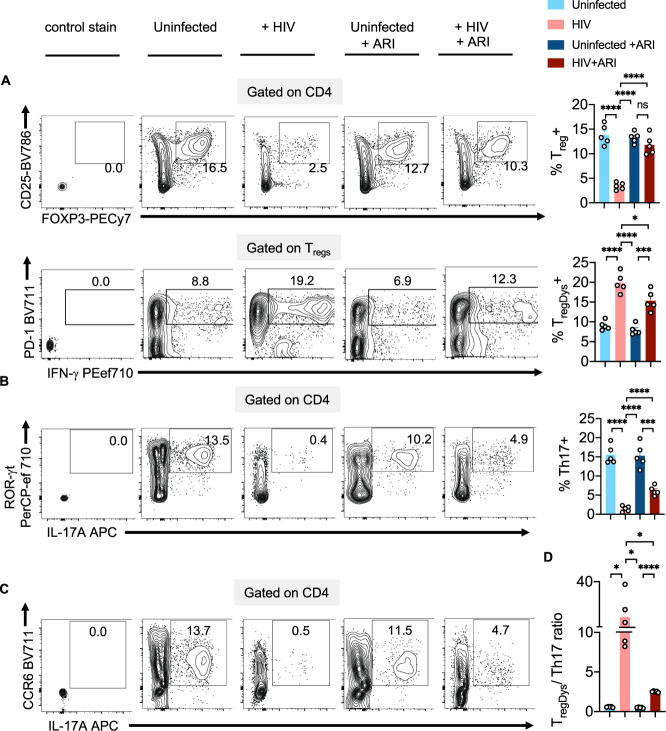
HIV infection causes Th dysregulation that sustained even after ARI treatment. TCR-stimulated HTOC was infected with HIV. Control cultures were not infected (Uninfected). In some cultures, ARI was added the next day to block subsequent rounds of infection. Cells were allowed to expand in the presence of TGF-β1 (10 ng/ml) and IL-2 (100 U/ml) for 6 days. **A** FOXP3 and CD25 expression (top) and PD-1 and IFN-γ expression (bottom) in CD4^+^ (CD3^+^CD8^neg^) FOXP3^+^ cells, showing the frequency of T_regs_ and T_regDys_. Expression of IL-17A and ROR-γt (**B**), and IL-17A and CCR6 (**C**) in CD4^+^ cells, showing the frequency of Th17 cells. Representative contour plots (left), statistical analyses of proportions of the cells from 5 independent experiments showing the frequency of these subsets (**A**, **B**, right). **D** Statistical analysis of T_regDys_/Th17 ratio based on the results in **A** and **B**. Results derived from five independent experiments are presented as mean values +/− SEM. **A**–**D** ****P < 0.0001; ***P < 0.0002; *P < 0.02; Two-tailed; Unpaired *t* test. Circles within box plots represent each replicate.

Next, we postulated that HIV-induced T_reg_ dysfunction in PLWH^4^ might involve the polyamine pathway. We employed the oral lymphoid system of TCR-activated human tonsil organoid cultures (HTOC) in vitro^5^. First, we examined ODC-1 expression in different TCR-stimulated CD4^+^ subsets in non-polarized and uninfected HTOC. ODC-1 is known to be upregulated in TCR-activated cells and highly expressed in in vitro-polarized Th1 and Th2 cells, but reduced in Th17 cells and T_regs_^5–7^. We compared CD25^+^ activated vs non-activated cells, IFN-γ^+^ Th1 cells vs IFN-γ^neg^ non Th1 cells, ROR-γt^+^IL-17A^+^ Th17 cells vs ROR-γt^neg^IL-17A^neg^ non Th17 cells, CD4^+^CD25^+^FOXP3^+^T_regs_ vs CD4^+^CD25^+^FOXP3^neg^ non T_regs_, and CD4^+^CD25^+^FOXP3^+^PD-1^+^IFN-γ^+^ T_regDys_, vs CD4^+^CD25^+^FOXP3^+^PD-1 ^neg^IFN-γ^neg^ non T_regDys_, in TCR-stimulated HTOC. CD25^+^ cells, Th1, Th17, and T_regs_ expressed higher ODC-1 levels compared to their counterparts (Supplementary Fig. 5). Consistent to a recent study^5^, Th1 cells showed increased ODC-1 expression than Tregs and Th17 cells. Our results also showed that T_regDys_ expressed significantly lower ODC-1 than non T_regDys_. These data suggested distinct differences in polyamine metabolism between CD4^+^ T cell subsets (Supplementary Fig. 5) and provided a rationale to examine polyamines in CD4^+^ T cells in the context of HIV infection. As shown previously, about 3- 7% HIV-GFP^+^ cells were observed upon HIV infection of these cultures^4,18^. HIV-1 infection causes significant depletion of IFN-γ and IL-17A^+^ effector Th subsets and results in the induction and expansion of dysfunctional PD-1^+^IFN-γ^+^FOXP3^+^ cells (T_regDys_)^4,18^. Because of the known role of Th17 cells in mucosal integrity, and the previous evidence of dysregulation of mucosal T_reg_/Th17 ratios in acute SIV infections^19^, we examined T_reg_/Th17 ratios in HIV-1-infected HTOCs. As the markers we used distinguished the subsets with relevance to ODC-1 expression (Supplementary Fig. 5), we employed the same to identify Th17 cells in HTOC. To assess the effects of HIV and anti-retroviral inhibitor (ARI) alone in cultures, we had these control cultures. To mimic HIV infection followed by anti-retroviral treatment in patients (Fig. 1), we added ARI efavirenz 24–36 h after HIV infection and allowed the cells to expand with ARI for 6–7 days. Similar to our previous findings^4,18^, T_regs_ showed significant cell death and were restored with ARI, efavirenz (Fig. 2A, top). After confirming T_regDys_ expansion during HIV infection that persisted in the presence of ARI (Fig. 2A, bottom), we assessed the frequency of Th17 cells by determining the expression of ROR-γt, CCR6, and IL-17A in CD4^+^ T cells. The cell numbers of Th subsets were also determined in these cultures (Supplementary Fig. 6A). Our results showed that HIV-1 infection led to a significant loss of Th17 cells and the ARI treatment post-HIV infection was not able to restore them in vitro (Fig. 2B, C). Alterations in the frequencies of these two subsets resulted in significant enhancement of T_regDys_/Th17 ratios during HIV infection and were sustained even after the ARI treatment (Fig. 2D). These results prompted us to determine the role of polyamines during HIV infection in the presence of ARI.

### HIV-1 upregulates ODC-1, NLRP3, EIF5A, EIF5A hypusination and polyamine synthesis in CD4^+^ T cells even in the presence of ARI

**Fig. 3:**
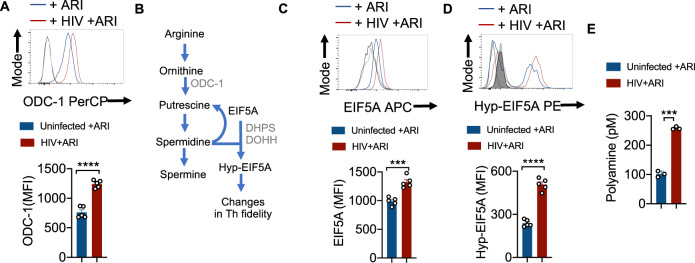
HIV-1 upregulates ODC-1, EIF5A, EIF5A hypusination and polyamine synthesis in CD4^+^ T cells even after ARI treatment. **A** HTOC were TCR-activated and infected with HIV. ARI was added the next day to block subsequent rounds of infection. Cells were allowed to expand in the presence of TGF-β1 (10 ng/ml) and IL-2 (100 U/ml) and ARI for 6 days as described in methods (*n* = 5 independent experiments). Representative flow cytometric data showing ODC-1 expression in CD3^+^CD8 negative cells (CD4+ T cells) (top) and statistical analyses of MFI from five independent experiments (bottom) are shown. **B** Key intermediate steps in polyamine synthesis. Cells were stimulated and processed for flow cytometry as in **A** for determining the expression of EIF5A (**C**) and hypusinated EIF5A in CD4^+^ T cells (**D**). Light and dark shaded gray histograms represent staining controls for EIF5A (**C**) and hypusinated EIF5A (**D**) respectively. Mean values +/− SEM; ****P < 0.0001; ***P < 0.0002; Two-tailed; Unpaired *t* test in **A**–**D**. **E** Fluorometric polyamine estimation in cellular lysates from cells stimulated as in **A** for 4 or 7 days (*n* = 3 independent experiments). Polyamine concentrations were normalized to cell numbers. Mean values +/− SD; ***P = 0.0002; Two-tailed; Welch’s *t* test. Circles within box plots represent each replicate.

To examine polyamine metabolism, we first focused on ODC-1 expression during HIV-1 infection in vitro. ARI was added 24–36 h after HIV infection and the cells were allowed to expand for 6–7 days. Consistent with the up-regulation of mRNA levels of *ODC1* and *EIF5a* in PLWH oral mucosa, we found that HIV-infected cells showed higher levels of this protein in the presence of ARI (Fig. 3A). Then we measured the expression of EIF5A and its hypusination that occurs downstream of ODC-1 activity (Fig. 3B). The expression of both EIF5A and hypusinated EIF5A were significantly upregulated in HIV-1-infected CD4^+^ cells in HTOC (Fig. 3C, D). HIF-1α is a metabolic checkpoint protein that can be induced by non-hypoxic stimuli such as TCR- and PI3-K pathways, which result in divergent functions in the pathogenesis of inflammatory diseases^20^. Given the increases in *HIF-1a* mRNA expression in PLWH (Supplementary Fig. 2), and the known roles of HIF-1α and polyamines in orchestrating Th lineage functions^5–7,21^, we speculated that HIF-1α may be linked to polyamine metabolism. However, our results showed that HIV-1 did not upregulate HIF-1α expression in CD4^+^ T cells in vitro (Supplementary Fig. 6B). Fluorimetry assay of polyamines showed that intracellular polyamines were also significantly elevated in CD4^+^ T cells in HIV infected cultures (Fig. 3E). By measuring polyamine levels in the supernatants of HIV infected cultures, we confirmed that increased intracellular polyamines was not due to decreased export to extracellular media (Supplementary Fig. 7). In summary, these results show that Th cells expanding in the presence of ARI post-HIV infection upregulate polyamine biosynthesis. These findings in oral mucosal lymphoid tonsillar cultures recapitulated the increased polyamine metabolism observed in the oral mucosa of HIV+ patients on therapy (Fig. 1A).

### HIV-1 activated caspase-1 and IL-1β are required for ODC-1 upregulation in CD4^+^ T cells

**Fig. 4:**
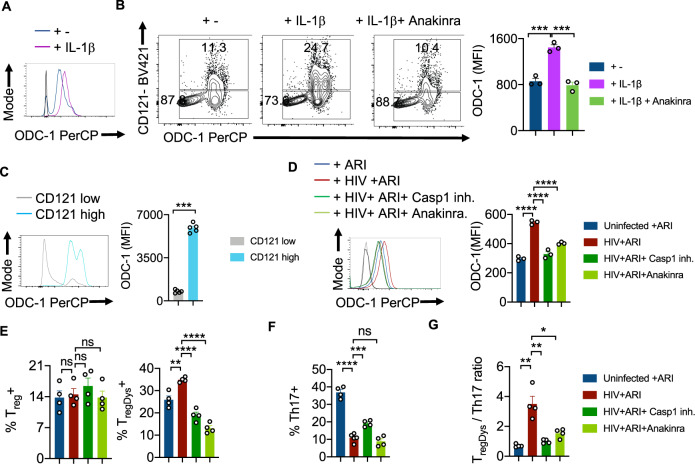
HIV-1 activated caspase-1 and IL-1β are required for ODC-1 expression in CD4^+^ T cells. HTOC cells were TCR stimulated with IL-1β (10 ng/ml) for 6 days in the presence or absence of Anakinra (10 μg/ml). **A** Histogram plot showing ODC-1 expression. **B** ODC-1 and CD121(IL-1R1) expression in CD4^+^ T cells (*n* = 3 independent experiments). **C** ODC-1 expression gating on CD121 high versus CD121 low cells (*n* = 5 independent experiments). Flow cytometry plots of unstained and FMO stain controls are provided in the Supplementary Fig. 9. **D**–**G** Purified tonsillar CD4^+^ T cells were TCR activated, infected with HIV and allowed to expand for 6-days post-infection in the presence of ARI, Efavirenz (50 nM). Caspase-1 inhibitor (VX-765, 250 nM) and Anakinra (10 μg/ml) were added as indicated, 36 h post-infection. ODC-1 expression (**D**), T_reg_ and T_regDys_ proportions (*n* = 3 independent experiments) (**E**), Th17 proportions (*n* = 4 independent experiments) (**F**), and T_regDys_/Th17 ratios (*n* = 3 independent experiments) (**G**) are shown. Light and dark gray histograms represent staining controls for ARI and HIV+ ARI conditions, respectively in **A** and **D**. Flow cytometry plots of these respective graphs are provided in Supplementary Fig. 11. Results are derived from three to four independent experiments and are presented as mean values +/− SEM. ****P < 0.0001; ***P = 0.0001–0.0002; **P < 0.002; *P 0.002–0.02; Two-tailed; Unpaired *t* test in **B**–**G**. Circles within box plots represent each replicate.

Previous studies by us and others have demonstrated the augmentation of NLRP3 signaling, activation of caspase-1 and elevated IL-1β expression in CD4^+^ T cells during acute and chronic HIV infection^4,22,23^. Based on ODC-1’s ability to regulate NLRP3 expression in macrophages^24^, and NLRP3’s function in activating caspase-1^25^, we hypothesized that NLRP3 may also be upregulated in HIV-infected HTOC CD4^+^ T cells. We confirmed that HIV-1 infection significantly induced NLRP3 in HTOC CD4^+^ T cells expanding in the presence of ARI (Supplementary Fig. 8). We then interrogated whether NLRP3/IL-1β signaling is involved in HIV-induced ODC-1 upregulation in the presence of ARI. IL-1β promoted the upregulation of ODC-1 in TCR- stimulated CD4^+^ T cells (Fig. 4A). Moreover, ODC-1 expression significantly correlated with the expression of IL-1Rα (CD121) on CD4^+^ T cells and was diminished by Anakinra, the IL-1β signaling inhibitor (Supplementary Fig. 9, Fig. 4B, C). To further elucidate the function of caspase-1 mediated cleavage and activation of IL-1β in inducing ODC-1, we employed VX-765 the caspase-1 inhibitor, and Anakinra. We infected the purified CD4^+^ cells from TCR stimulated HTOC, infected with HIV-1 in the presence or absence of inhibitors, and treated them with ARI, 24–36 h post-infection. Flow cytometry analysis revealed that both VX-765 and Anakinra significantly downregulated ODC-1 expression in HIV-1-infected cells(Fig. 4D). Neither NLRP3 inhibition nor HIF-1α inhibition directly modulated ODC-1 expression or increase in T_regDys_ in HIV-infected cultures (Supplementary Fig. 10A, 10B). While Caspase-1 and IL-1β blockade did not affect T_reg_ frequencies, they significantly decreased the T_regDys_ frequencies (Fig. 4E, Supplementary Fig. 11). On the other hand, Caspase-1 inhibition through VX-765 partially restored Th17 cell frequencies, but blocking of IL-1β did not impact HIV-1 mediated Th17 loss (Supplementary Fig. 11, Fig. 4F). Further analysis showed that Th17 cell loss was due to the cell death triggered by HIV-1, only partially restored by ARI (Supplementary Fig. 12). Caspase-1 involvement in Th17 cell loss suggests that cell death is pyroptotic. These data indicate that during HIV infection, caspase-1 is critical to 1) IL-1β release and T_regDys_ increase by ODC-1 up-regulation and 2) Th17 cell loss through pyroptotic cell death. Together, caspase-1 and IL-1β drive the T_regDys_/Th17 skewing during HIV infection (Fig. 4G). These data position caspase-1 and IL-1β at the juncture of polyamine pathway dysregulation, cell death mechanism, and Th fate alteration induced by HIV-1.

### HIV infection mediated Th dysregulation is dependent on ODC-1

**Fig. 5:**
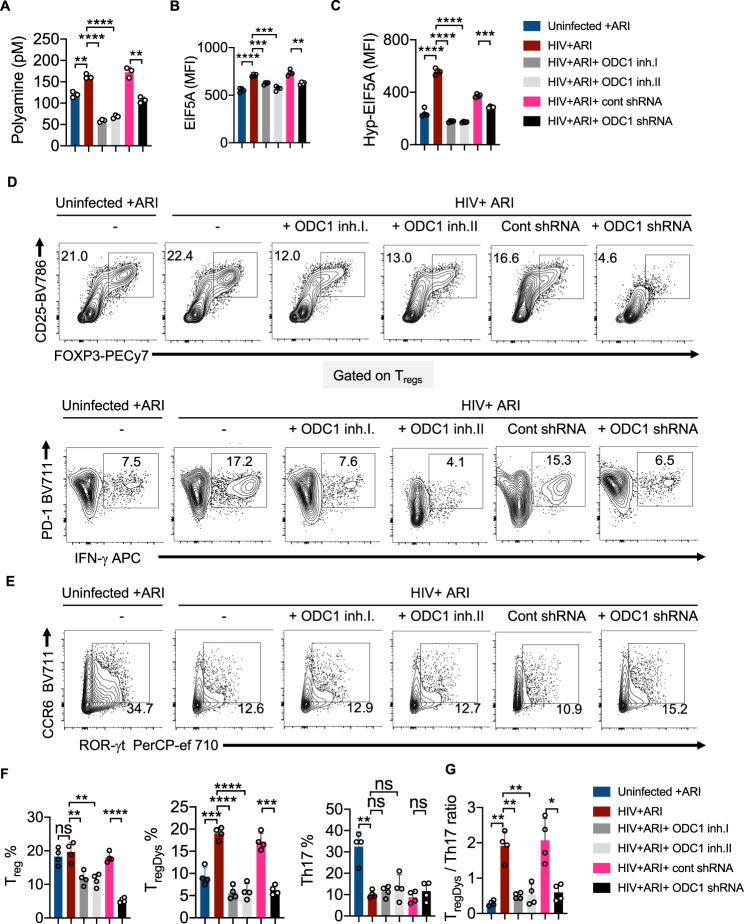
HIV-1 infection associated Th dysregulation is dependent on ODC-1 activity. HTOC CD4^+^ T cells were TCR activated and infected with HIV. ARI (Efavirenz; 50 nM), DFMO (ODC-1 inhibitor I; 2.5 mM), POB (ODC-1 inhibitor II; 100 μM), Control shRNA lentiviral particles-A with polybrene (2 μg/ml), and ODC1 shRNA lentiviral particles with polybrene (2 μg/ml) were added as indicated, 36 h post-infection. **A** Fluorometric polyamine estimation in cell lysates on day 6 post- infection. *n* = 3 independent experiments; Mean values +/− SD; **P < 0.003; ****P < 0.0001; Two-tailed; Welch’s *t* test. **B** Statistical analysis of EIF5A expression (MFI), and **C** Statistical analysis of hypusinated EIF5A expression (MFI) from three experiments. Flow cytometric overlays are provided in Supplementary Fig. 13. **D** Contour plots showing FOXP3 and CD25 (T_regs_) expression in CD4 cells (above) and PD-1 and IFN-γ (T_regDys_) in FOXP3^+^cells (below). **E** Contour plots showing ROR-γt and CCR6 expression (Th17) in CD4 cells. Quantification of T_reg_, T_regDys_, Th17 (**F**), and T_regDys_/Th17 ratios (**G**) based on the data from **D** and **E**. Data are representative of four (*n* = 4) independent experiments and are presented as mean values +/− SEM. ****P < 0.0001; ***P = 0.0001; **P < 0.005; *P < 0.02; Two-tailed; Unpaired *t* test in **A**–**C** and **F**–**G**. Circles within box plots represent each replicate.

To confirm that ODC-1 activity underlies polyamine synthesis and T_reg_ dysregulation caused by HIV-1, we infected HTOC CD4^+^ T cells in the presence or absence of ODC-1 inhibition using Difluoromethylornithine (DFMO) (ODC-1 inhibitor I), N-(4-Pyridoxyl)-Ornithine(BOC)-OMe (POB)(ODC-1 inhibitor II), and ODC-1 shRNA lentiviral particles. Using the polyamine fluorometric assay, we first confirmed that ODC-1 inhibition diminishes intracellular polyamines (Fig. 5A). Consistent with the requirement of ODC-1’s activity in promoting EIF5A hypusination, inhibition of ODC-1 significantly downmodulated the expression of both EIF5A and hypusinated EIF5A in HIV-1 infected CD4^+^ cells in HTOC (Fig. 5B, C, Supplementary Fig. 13). As expected, ARI restored the T_reg_ levels to uninfected, but ODC-1 inhibition further decreased T_regs_ moderately (Fig. 5D, top, 5F). While HIV-1 infection boosted the frequency of T_regDys_, blockade of ODC-1 activity significantly diminished T_regDys_ to the levels in uninfected cultures (Fig. 5D, bottom, F). ODC-1 inhibition did not affect the Th17 depletion caused by HIV, (Fig. 5E, F), but it significantly restored the T_regDys_/Th17 ratios to the uninfected levels in CD4 T cells (Fig. 5G). These findings strengthen the idea that ODC-1 is required for the enrichment of intracellular T cell polyamine levels and Th fate skewing caused by HIV-1.

### HIV infection mediated polyamine synthesis is dependent on ODC-1

**Fig. 6:**
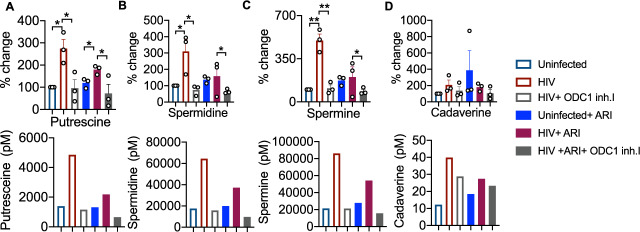
HIV-1 infection associated polyamine increase is dependent on ODC-1 activity. HTOC CD4^+^ T cells were TCR activated and infected with HIV. ARI and ODC-1 inhibitor I were added as indicated, 36 h post-infection. Quantification of putrescine (**A**), spermidine (**B**), spermine (**C**), and cadaverine (**D**) relative to the uninfected controls are shown (top). Thermospermine was not detected. Means with standard deviation error bars from three independent experiments are shown. *n* = 3 independent experiments. Unpaired *t* test one-tailed with Welch’s correction; *P values 0.0126–0.0243, **P values 0.0038–0.0085 in **A**–**D**. Circles within box plots represent each replicate. Absolute levels (normalized to the input cell numbers) of the respective polyamines from one of the three independent experiments (bottom).

To further determine the role of ODC-1 in polyamine synthesis in CD4^+^ cells in the context of HIV infection, we performed the targeted assessment of specific polyamines in cells in the presence or absence of ODC-1 inhibition. We quantified putrescine, spermine, spermidine, cadaverine and thermospermine in cellular lysates using LC-MS as described in methods^26^. HIV-1 significantly upregulated putrescine levels both in the presence and absence of ARI. ARI partially reduced putrescine in HIV infected cultures (Fig. 6A). Spermidine and spermine were also significantly elevated during HIV infection, but ARI restored them almost to uninfected levels (Fig. 6B, C). Cadaverine was unaffected by HIV infection (Fig. 6D). Importantly, ODC-1 inhibition significantly diminished HIV-induced intracellular increases in putrescine, spermidine, and spermine, showing the critical function of ODC-1 in enhancing polyamine levels during HIV-1 infection (Fig. 6A–C).

### HIV-induced skewing of T_regDys_/Th17 ratios requires polyamine synthesis and exogenous polyamines cause Th dysregulation

**Fig. 7:**
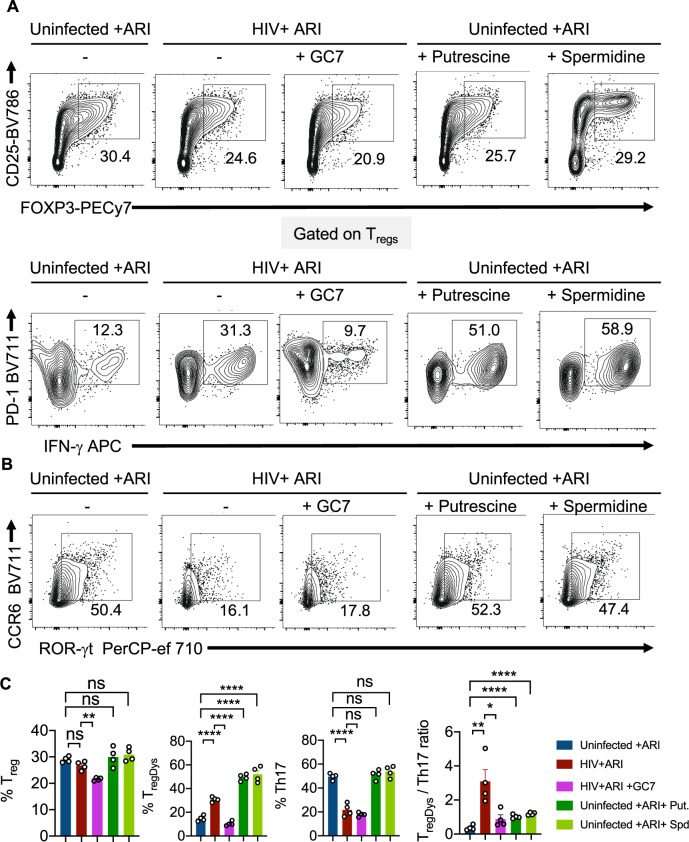
HIV-induced skewing of T_regDys_ /Th17 ratios requires polyamine synthesis. HTOC CD4^+^ T cells were stimulated as described in the methods. Some cultures were HIV-infected with or without GC7 (10 μM). Other uninfected cultures were treated with Putrescine dihydrochloride (100 μM) or Spermidine (1 mM). **A** Contour plots showing the percentage of T_regs_ (above) and PD-1^hi^IFN-γ^+^ cells in FOXP3^+^ population (T_regDys_) (below). **B** Contour plots showing ROR-γt and CCR6 expressing CD4^+^ T cells (Th17). **C** Quantification of T_regDys_/Th17 ratios as determined by flow cytometry analyses in **A** and **B**. Results represent four experiments with similar results and are presented as mean values +/− SEM. ****P < 0.0001; ***P = 0.0004; **P = 0.007; *P < 0.02; Two-tailed; Unpaired *t* test in **C**. Circles within box plots represent each replicate.

We next determined whether polyamine enrichment and EIF5A hypusination could have contributed to HIV-mediated increase in T_regDys_/Th17 ratios. To corroborate the effect of polyamines and EIF5A hypusination independent of HIV-1 infection, we added exogenous putrescine hydrochloride, spermidine, and GC7, a DHPS inhibitor. Analogous to ODC-1 inhibition, GC7 also precipitously lessened the T_reg_ and T_regDys_ frequencies (Fig. 7A, top and bottom, first two panels in Fig. 7C), but did not restore Th17 depletion (Fig. 7B, 3^rd^ panel in Fig. 7C) in HIV-infected HTOC CD4 cultures. While exogenous polyamines dramatically increased the frequency of T_regDys_, they did not temper the proportions of Th17 cells in uninfected cultures (Fig. 7A, B; last two panels and Fig. 7C). Although exogenous polyamines did not increase the T_regDys_/Th17 ratios as dramatically as HIV-1, they significantly increased them (Fig. 7C, last panel). Although exogenous polyamines did not increase T_reg_ frequencies or lowered Th17 proportions, by simply increasing T_regDys_ frequencies, they still significantly increased the T_regDys_/Th17 ratios. A closer examination of the viability of non T_regs_, T_regs_, and T_regDys_ revealed that ODC-1 inhibition leads to cell death (Supplementary Fig. 14). However, ODC-1 inhibition and GC-7 caused significantly more cell death in non T_regs_ than in T_regs_ or T_regDys_. Similarly, spermidine also caused loss of viability in non T_regs_ but not in T_regs_ or T_regDys_. These data show that these inhibitors did not reduce T_regDys_ by causing their cell death. Moreover, exogenous polyamines did not alter the viability of T_regs_ or T_regDys_, ruling out the possibility that polyamines increase the proportions of T_regDys_ simply by increasing their viability. Because polyamines and GC7 have been implicated in autophagic cell death^27^, we further determined the expression of microtubule-associated proteins 1A/1B light chain 3B (LC3B), an autophagic protein in CD4^+^ T cells. While HIV infection, ARI, ODC-1 inhibitor I, or spermidine did not alter the protein levels, cell treated with GC7 showed elevated levels of LC3B (Supplementary Fig. 15). By using LY294002, an autophagic sequestration inhibitor, we further found that ODC-1inhibition and GC7 reduced T_regDys_ proportions independently of autophagic cell death during HIV infection (Supplementary Fig. 16). These data highlighted the autophagic-independent effect of polyamines and EIF5A hypusination on T_regDys_ cells and significantly heightening the T_regDys_/Th17 ratios in HTOC during HIV-1 infection.

### Exogenous addition of polyamines causes EIF5A upregulation, EIF5A hypusination leading to induction of T_regDys_ cells and their proliferation

**Fig. 8:**
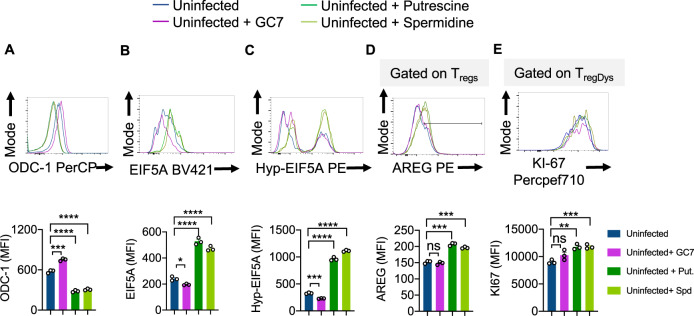
Exogenous addition of polyamines causes EIF5A upregulation, EIF5A hypusination leading to induction of T_regDys_ cells and their proliferation. HTOC CD4^+^ T cells were TCR stimulated in the presence of GC7 (10 μM), Putrescine dihydrochloride (100 μM), or Spermidine (1 mM) without infection. Flow cytometry histogram overlays (top) and statistical quantification of MFIs (bottom) based on the flow cytometry analysis of ODC-1 (**A**), EIF5A (**B**), Hypusinated-EIF5A (**C**), AREG (**D**), and KI-67 (**E**) staining 6 days after stimulation. Means from triplicate experiments are plotted. ****P < 0.0001; ***P < 0.0006; **P = 0.002; *P < 0.01; Two-tailed; Unpaired t test in **A**–**E**. Circles within box plots represent each replicate.

To further corroborate the effect of polyamines on Th cells, we evaluated the expression of ODC-1, EIF5A, and hypusinated-EIF5A in uninfected CD4^+^ T cells. Polyamines enhanced the expression of EIF5A, and hypusinated-EIF5A but significantly downmodulated ODC-1 expression in CD4^+^ T cells (Fig. 8A–C). To determine whether polyamines synthesized during HIV infection and exogenous polyamines induce FOXP3^+^PD-1^+^IFN-γ^+^T_regDys_ or enhance IFN-γ in general in all CD4^+^ cells, we examined the expression of IFN-γ in non T_reg_ CD4^+^ cells. As we showed previously, HIV-infection in fact reduced IFN-γ in non T_reg_ CD4^+^ cells and ARI partially restored IFN-γ^+^ cells (Supplementary Fig. 17). Neither ODC-1 inhibition nor GC7 affected IFN-γ expression in non T_regs_, showing that they reduced T_regDys_ frequencies without regulating IFN-γ in CD4^+^ T cells during HIV infection. Corroborating these results further, spermidine rather downmodulated IFN-γ in uninfected non T_reg_ CD4^+^ cells. These data show that polyamines do not induce T_regDys_ by up-regulating IFN-γ in FOXP3^+^ cells. On the contrary, a closer examination of all PD-1^+^IFN-γ^+^CD4^+^ cells (Th1 like cells) revealed that HIV-1 infection and exogenous spermidine induced FOXP3 expression in them (Supplementary Fig. 18). ODC-1 blocking and GC7 inhibited the FOXP3 induction during HIV-1 infection. Taken together, polyamines upregulated during HIV infection appear to inhibit IFN-γ in CD4^+^ cells or may even cause cell death of IFN-γ^+^CD4^+^ cells causing dysregulation of Th1-like cells. Additionally, they also induce FOXP3 in a proportion of these Th1-like cells thus promoting T_regDys_ cells and Th infidelity. We have previously shown that T_regDys_ characteristically express high levels of Amphiregulin (AREG)^4^. Here we found that polyamines also upregulated AREG moderately but significantly in T_regs_ (Fig. 8D). Consistent with their ability to promote proliferation, polyamines also enhanced T_regDys_ expansion as shown by increased KI-67 expression (Fig. 8E). Together, these data uncover the function of excessive polyamines in causing Th infidelity and involves downmodulation of IFN-γ^+^ CD4^+^ cells, induction of FOXP3 in dysregulated Th1-like cells and proliferation of T_regDys_ cells. These results also revealed a potential negative-feedback loop effect of polyamines in repressing ODC-1 expression during HIV infection (Fig. 8A).

### Increased T_regDys_/Th17 ratio correlates with polyamine levels in oral mucosa of PLWH

**Fig. 9:**
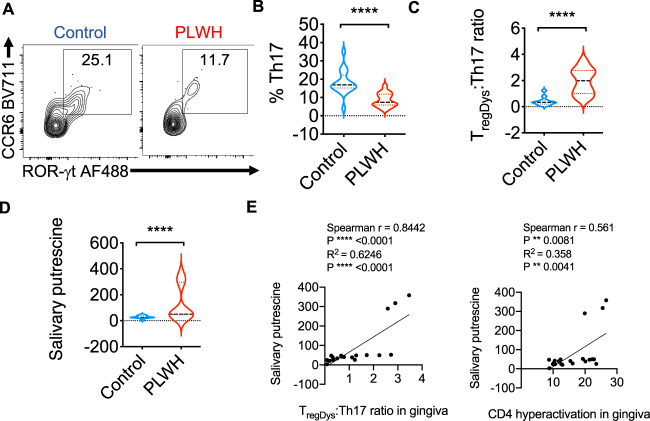
HIV+ patients have skewed T_regDys_/Th17 ratios and CD4^+^ T cell hyperactivation correlating with salivary putrescine in the oral mucosa. Gingival mucosal cells from healthy controls (*n* = 19) and PLWH (*n* = 30) were processed for flow cytometry ex vivo. **A**, **B** Th17 cells were quantified by examining the expression of ROR-γt and CCR6 in CD4^+^ T cells. Contour plot (**A**), and statistical quantification (**B**), show Th17 proportions. The increase in T_regDys_ proportions and Th hyperactivation (CD38^+^HLADR^+^CD4^+^T cells) in this PLWH cohort were previously published^4^. **C** Statistical quantification of T_regDys_ /Th17 ratios as determined by flow cytometry analyses. **D** Statistical quantification of salivary putrescine using LC-MS in healthy controls (*n* = 11) and PLWH (*n* = 10) with known T_regDys_ /Th17 ratios as determined by flow cytometry analyses. Median values ± SEM are plotted. (Mann–Whitney *U* test ****P < 0.0001; **P = 0.007; Two-tailed in **B** and **C**. Circles within box plots represent each replicate. **E** Correlation between gingival T_regDys_/Th17 ratios and salivary putrescine (*n* = 21) (left), and Correlation between gingival CD4 hyperactivation and salivary putrescine (*n* = 21)(right). *P* values from both correlation and regression analysis are provided.

Although the oral mucosa of HIV+ patients exhibits a significantly higher frequency of T_regDys_ contributing to Th hyperactivation (CD38^+^HLADR^+^CD4^+^T cells)^4^, the fate of Th17 cells has not been evaluated previously. Based on the results from HIV infection of HTOC CD4^+^ T cells in vitro (Fig. 2), we hypothesized that Th17 cells may also be reduced in the oral mucosa of HIV+ patients even after cART therapy. To test this notion, we evaluated the frequency of Th17 cells, as defined by the expression of CD3^+^CD4^+^CCR6^+^ROR-γt in oral gingival mucosa. Although ~25% of CD4^+^ cells from healthy controls conformed to the Th17 phenotype, only about 11% of Th17 cells were found in HIV+ patients (Fig. 9A). Concurring with in vitro HIV infection results, we found that PLWH had a reduced percentage of Th17 cells than un infected control individuals (Fig. 9B). Then we plotted the T_regDys_/Th17 ratios based on the previously published^4^ T_regDys_ proportions in this cohort. These results showed significantly increased T_regDys_/Th17 ratios in the oral mucosa of PLWH (Fig. 9C). Although ODC-1 expression did not correlate with T_regDys_/Th17 ratios (Supplementary Fig. 19), putrescine levels were significantly elevated in saliva (Fig. 8D) and showed a significant positive correlation with T_reg_/Th17 ratios and Th hyperactivation in the oral mucosa (Fig. 8E). Altogether, these findings from oral gingival mucosal cells of PLWH corroborate the results from HTOC HIV infections and highlight the role of aberrant polyamine pathway in determining mucosal Th fates and persistent immune hyperactivation during chronic viral infections.

## Discussion

Using metabolomics, transcriptomics, flow cytometry, and functional approaches, this study uncovers the pleiotropic effects of excessive polyamines on mucosal T cell functions during a chronic viral infection (Graphical abstract; Supplementary Fig. 20). We performed an integrated mapping of metabolomic and transcriptomic changes that occurred in the oral mucosa and persisted even after therapy in PLWH. Cellular metabolism is a prime regulator of both immune cell proliferative capacity and effector functions^28^. Analysis of saliva metabolites and transcriptomics of oral mucosal cells in tandem identified several key pathways that were altered in response to infection. Excessive putrescine correlated with the tipping of Th subset changes and CD4 hyperactivation in oral mucosa in the context of treated HIV infection in PLWH. Using HTOC cultures, we also found that HIV-1 upregulates polyamines in CD4 T cells and results in induction and secondary proliferation of T_regDys_ in vitro (Figs. 6 and 8E). We found that blocking subsequent rounds of HIV-1 infection and cell death with ARI did not normalize the putrescine or the induction and proliferation of T_regDys_. This is consistent with our previous finding that ARI does not reverse Th dysfunction completely^4^.

Amino acid metabolism was significantly enriched at both the individual metabolite level and in-combination with transcriptomic analysis. Notably, alterations in nitrogen metabolism were evident in the increased expression of enzymes involved in polyamine synthesis that occurred in conjunction with decreased ornithine levels and increased putrescine in saliva. Polyamines are polycations that regulate cell growth, protein translation and stress responses^29,30^. Also, excessive polyamines and EIF5A over-expression are associated with cancers^31^. Polyamine-EIF5A axis could have a broader role in general mucosal dysfunction as well as cancer predisposition in HIV+ patients, but this study focused only on its role in Th dysfunction. Recent studies have shown that polyamines and related metabolites regulate Th cell responses^5,6,17,32^. It is well-known that CD8^+^ T cells upregulate ODC-1 and increase polyamine biosynthesis, which is required for their cytolytic function^33^. Putrescine and arginine, both of which show significant upregulation in saliva in HIV patients (Fig. 1C), have been shown to suppress the development of the cytolytic function of CD8^+^ T cells in mixed lymphocyte cultures^34^. Polyamine biosynthesis has also previously been shown to enhance T cell activation^35^, which is dependent on MYC-induced changes in glutamine and glucose utilization, although we did not observe any changes in these pathways in PLWH. While our findings here position polyamines as contributors to pathogenic Th inflammation in the mucosa, the impact of excessive polyamines on CD8^+^ T cell functions and tumorigenesis remain to be seen.

The overall mucosal immune dysfunction in PLWH was also evident in altered lipid and tryptophan metabolism in the oral mucosa. The synthesis of inflammatory lipids from arachidonic acid is a hallmark of tissue inflammation^36^. HIV-infected patients have significant increases in both arachidonic acid itself as well as two inflammatory eicosanoids, 12-HETE and 5-HPETE. Changes in the levels of these inflammatory lipids are also reflected in transcriptional alterations in the enzymes (CYP4F3 and ALOX5) that synthesize them. 12-HETE production leads to oxidative stress at the sites of inflammation^37^, which may exacerbate virus-induced pathology. Also, alanine aminotransferase (GPT2) is significantly decreased during HIV infection (Fig. 1C). This enzyme catalyzes the transfer of an amino group from alanine to α-ketoglutarate, producing pyruvate and glutamate. Exogenous alanine uptake is essential to T cell activation^38^ and impairment of alanine utilization during HIV infection may result in reduced effector functions in the oral mucosa. Although alterations in these pathways implicate critical alterations in the mucosal immune system of HIV+ patients, the exact roles of these pathways in the context of the signaling components described here during chronic viral infection remain to be defined.

Ingested food and resident microbiota are also significant sources of polyamines^39^. A potential possibility of oral microbial dysbiosis in also contributing to polyamines in PLWH is intriguing and will be addressed in future studies. Aberrations in the polyamine-EIF5A-hypusine axis have also been shown to modulate TCA cycle re-wiring, mitochondrial respiration, epigenetic re-modeling in macrophages and T cells^5,40^. However, our results showed that polyamine enrichment did not coincide with TCA cycle dysregulation in oral mucosa of HIV+ patients (Supplementary Figs. 1 and 2). Whether polyamine-mediated T_regDys_ accumulation is accompanied by epigenetic modifications of cytokine loci in addition to proliferation of dysregulated T cells remains to be seen (Graphical abstract; Supplementary Fig. 20).

Our study focused on the pathway upstream to polyamine biosynthesis and the data identify caspase-1 mediated IL-1β release as a central event in HIV-1 induced ODC-1 up-regulation in Th cells (Fig. 4). These results are consistent with the ability of IL-1 to induce ODC-1 activity^41,42^. Higher expression of IL-1 receptor in T_regDys_ than in other non T_reg_ CD4^+^ cells^4^ (Fig. 4), may explain why IL-1β-mediated upregulation of ODC-1 may promote T_regDys_ cells during HIV infection (Fig. 5D). Thus caspase-1 and IL-1β pathways appear to be central in affecting polyamine metabolism dependent immune-dysfunction during viral infections (Supplementary Fig. 20). ODC-1 expression analysis in various tonsillar CD4^+^ subsets showed the least expression of the protein in T_regDys_ in a non-infection setting (Supplementary Fig. 5). Given the dominant role of ODC-1-polyamine-EIF5A hypusination axis in promoting T_regDys_ induction and proliferation during HIV-1 infection, we anticipated elevated levels of ODC-1 expression in T_regDys_. Lower ODC-1 levels could be due to negative feedback mechanism of excessive polyamines resulting in ODC-1 degradation or antizyme binding in T_regDys_^43^ (Fig. 8A). More work and fresh clinical samples are needed to determine the expression of ODC-1, EIF5A, Hyp-EIF5A specifically in Th17, T_reg_, and T_regDys_ in PLWH to achieve a complete understanding of how ODC-1-polyamine-EIF5A hypusination and the auto-regulatory loop impacts Th cells during viral infections.

Many cellular and cytokine changes in HIV-infected HTOC cultures recapitulated oral mucosa of PLWH and there were striking similarities between these systems in terms of T_regDys_ enrichment, Th17 loss not restored by cART, and increases in putrescine. However, acute HIV-infection system does not completely recapitulate some of the features found in PLWH. For example, 1) PLWH on cART show increased frequencies of CD4^+^CD25^+^FOXP3^+^T_regs_ in oral mucosa compared to healthy controls. However, in vitro HIV infection leads to CD4^+^CD25^+^FOXP3^+^T_reg_ cell death, although to a lesser extent than in other CD4^+^ T cells. While this caspase dependent death is reversed by ARI, T_reg_ proportions don’t exceed uninfected cultures. 2) ODC-1 levels do not correlate with T_reg_/Th17 ratio in PLWH (Supplementary Fig. 19), while ODC-1 inhibition significantly diminishes T_reg_/Th17 ratio in cultures, 3) CD4^+^ T cells from PLWH on cART show increased HIF-1α expression, but in vitro infection doesn’t upregulate HIF-1α in CD4^+^ T cells. Some of these differences could be mainly contributed by the effects of mucosal cells *e.g* epithelial cells found in gingiva and not found in HTOC cultures. While HTOCs provide a rich lymphoid environment with a wealth of CD4^+^ T cells, short-term cultured cells that are also susceptible to cell death may not fully recapitulate the mucosal CD4^+^ T cells in PLWH on long-term cART. We are establishing long-term cART cultures to address this concern in the future. With regards to inhibition studies, pharmacological inhibitors allow us to block targets in short-term primary cultures, but they often have off-target effects or cell-death effects and can confound results. Aware of these caveats, we have interpreted the results with much caution and used other approaches to examine the off-target effects. Also, results were evaluated in tandem with the supporting data from cells derived ex vivo from patients.

With regards to Th17 cells, Wagner et al., have shown that ODC-1 and SAT-1 regulate Th17 lineage proteins^6^. Therefore, we expected that ODC-1 and SAT-1 would play important roles in regulating Th17 cells, considering these proteins were also upregulated in PLWH. However, in line with previous findings, acute HIV-1 induced caspase-1 activity leads to Th17 inflammatory cell death^44^ and is independent of ODC-1/polyamine synthesis. Thus, Th17 regulation in the context of pyroptosis is unique to HIV infection and is significantly different from in vitro naïve cell differentiation in the context of Th17 polarizing cytokines and pathogenic Th17 cells in other settings. The dominant pyroptotic effect of HIV-1, independent of polyamine axis might explain why ODC-1 blocking alone post-HIV infection was unable to restore Th17 cells (Fig. 5E). Although NLRP3 is upregulated during HIV infection, it does not regulate polyamine metabolism. However, whether ODC-1 promotes NLRP3 expression and sustained NLRP3 upregulation contributes to further enhancement of caspase-1 activity and IL-1β levels remain to be seen. High NLRP3 expression independent of polyamine axis may also further contribute to Th17 cell death in chronic infection (Graphical abstract; Supplementary Fig. 20). Because caspase-1 inhibition or ARI did not restore Th17 cells completely, we speculate that HIV-1 could also be involved in transcriptional regulation of IL-17A cytokine in CD4^+^ T cells. These data also explain the reduced proportions of Th17 cells even after the cART treatment (Fig. 9A, B). It is tempting to speculate that mucosa may take a double hit due to T_reg_ dysfunction and Th17 impairment, which could lead to loss of barrier integrity and oral dysbiosis. Thus, chronic viral infections involving increases in IL-1β may trigger perturbations in polyamine synthesis and Th fate decisions, which may further fuel pathogenic inflammation in the mucosa. Taken together, dissection of immune dysfunction mechanisms in PLWH has provided us deeper insights into processes by which viruses may contribute to chronic inflammation by affecting the versatility of the immune system and manipulating polyamine metabolic flux in the mucosa. These findings may also lead to therapeutic immunomodulation approaches involving targets in polyamine metabolism.

## Methods

### Human samples

Human samples (gingival biopsies and saliva) were obtained with informed consents from healthy and HIV+ (PLWH) cohorts under a protocol approved by the University Hospitals Cleveland Medical Center Institutional Review Board, complying with all relevant ethical regulations^4^. Participants of both sexes were enrolled. The characteristics of enrolled participants for obtaining gingival biopsies and saliva are described in Supplementary Table 1. Gingival biopsies were processed fresh for flow cytometry. Discarded palatine tonsils were obtained from tonsillectomy surgeries performed at University Hospitals Cleveland Medical Center through the Histology Tissue Procurement Facility following a separate IRB-approved protocol (Non-Human research). A single-cell suspension of gingival tissues and tonsils was prepared by Collagenase 1A digestion (0.5 mg/ml; Sigma C9891), with subsequent Ficoll-Paque PLUS (GE17-1440-02; Millipore Sigma) centrifugation at 900 g and washing with PBS. Tonsil cells were processed fresh for HTOC cultures. Some tonsil cells were processed fresh for CD4 cell purification for cell culture or flow cytometry. Saliva samples were collected in sterile tubes and stored at −80 °C until processing for metabolome and ELISA analyses.

### HTOC cultures and HIV infection

HTOC cultures were setup by resuspending collagenase-digested tonsillar cells at 1 million/ well in the upper well of 24 well transwell culture plates^4,45^. They were plated with α-CD3 (1 μg/ml) and α-CD28 (1 μg/ml) TCR activating antibodies, TGF-β1 (10 ng/ml) and IL-2 (100 U/ml) for 24–36 h before infection at least in triplicate wells^4,46^. For infections, cells in 200 µl volumes were spinoculated with replication competent HIV X4-tropic NL43-GFP-IRES-Nef or HIV-NLGNef (~1.7 ng), a recombinant virus with NL4-3 backbone expressing Green Fluorescent Protein (GFP) and Nef on a bicistronic transcript was used along with indicated inhibitors^4,47,48^. Confirmatory experiments were performed using both X4- and R5-tropic viruses^48^. The R5-tropic virus was created by replacing the Env in NL43-GFP-IRES-Nef from NLAD8, an NL43 construct containing CCR5-tropic HIV-1 ADA envelope (NIH AIDS Reagent Program)^47,49^. After allowing an initial round of infection for 24–36 h post-infection, Efavirenz (ARI) (SML1284-1ML; Millipore Sigma; 50 nM) was added to cultures. Then cells were expanded for 4–7 days before performing flow cytometry and other assays. Complete RPMI-1640 (Hyclone) supplemented with 10% human serum, 100 U/ml penicillin, 100 µg/ml streptomycin, 2 mM glutamine, 25 mM HEPES and 1 mM sodium pyruvate was used for cell cultures. Cells were rested for 24–36 h in a medium without TCR activation in select experiments. When indicated, purified CD4^+^ T cells were used in some experiments.

### Cell culture reagents and inhibitors

T cell receptor (TCR) stimulating antibodies for CD3 (HIT3a) and CD28(CD28.2) that were used in cell culture were purchased from BD Biosciences and Thermofisher Scientific respectively. Recombinant TGF-β1 was purchased from R&D systems and BioLegend. Nigericin was purchased from Invivogen. Recombinant IL-2, IL-1β, and IL-33 cytokines were purchased from BioBasic Inc. (Amherst, NY). IL-1 receptor antagonist Anakinra was added during HIV infection and was a kind gift from Dr. Su at NIAID, NIH. HIF-1α, ODC1, NLRP3 inhibitors, Putrescine dihydrochloride (100 μM), and Spermidine (1 mM) were also added during HIV infection when indicated. For CD4^+^ cell purification, CD4 T cell kits were purchased from Stem Cell Technologies (Vancouver, Canada). Cotinine, AREG, IL-1β, and IL-6 ELISA kits were from Boster Bio (Pleasanton, CA). Lentiviral transduction was performed using control or ODC-1 sh-RNA on day 1 after initial TCR activation and a day before HIV infection. Indicated ODC-1 inhibitors and control and ODC-shRNA lentiviral particles were purchased from Santa Cruz Biotechnology. Transduction was performed as per the manufacturer’s instructions. HIF-1α inhibitors and NLRP3 inhibitor (MCC950) were purchased from MedChemExpress. VX-765, a caspase-1 inhibitor was purchased from InvivoGen. Putrescine dihydrochloride and Spermidine were purchased from Millipore Sigma.

### Fluorochrome antibodies, staining and flow cytometry

Flow cytometry antibodies for ROR-γt (AFKJS-9), IL-1β (CRM56), CCR6 (R6H1), IL-17A(eBio64DEC17), CD25 (M-A251), Ki-67 (SolA15), HIF-1α (Mgc3), CD4 (OKT4), CD45(HI30), CD8 (RPA-T8), IFN-γ (4S.B3), FOXP3(236A/E7), AREG (AREG559), HLADR, and phospho-caspase 1 (Ser376) were all purchased from Invitrogen/Thermofisher Scientific. LC3B (IC9390R) and NLRP3 (768319) antibodies were purchased from R&D systems and BIOSS. CCR6, CD279 (PD-1) (EH12.1), BCL-2(Bcl-2/100), and IL-1R1(hIL1R-M1) antibodies were from BD Biosciences. ODC-1 antibody was purchased from Novus Biologicals. EIF5A and Anti-hypusine antibodies were purchased from Thermofisher Scientific and Millipore Sigma respectively. Surface receptors were first stained in PBS/BSA, followed by live-dead viability staining before fixation and intracellular staining. For FOXP3 and other intracellular proteins, the FOXP3 fixation-permeabilization set (Thermofisher Scientific) was used. Appropriate unstimulated, unstain, isotype, secondary antibody alone, single stain, and FMO controls were included in all the preliminary and confirmatory experiments, based on which the gates were determined in the analysis. Also, as an additional control for tissue cells, PBMCs were used. Cultures were additionally re-stimulated with PMA (50 ng/ml) and Ionomycin (500 ng/ml) for 4 h, with brefeldin-A (10 µg/ml) added in the last 2 h, before intracellular cytokine staining. For p-Caspase-1 staining, the cells were washed, fixed, and stained with a Phosflow staining kit (BD Biosciences) using the manufacturer’s protocol. The primary antibodies used for surface and intracellular staining were used at 1:50-1:200 dilutions respectively, or according to the manufacturer’s recommended dilution. Appropriate flow cytometry secondary antibodies such as secondary donkey anti-mouse IgG- BV421 (for EIF5A staining) and anti-rabbit PE (for hypusine staining) were purchased from Jackson Immunoresearch or Invitrogen/Thermofisher. The secondary antibodies for flow cytometry were used at a dilution of 1:500-1:800. BD Fortessa cytometers (BD FACSDiva software ver.7) and FlowJo 9.8 − 10.7.1 software versions for data acquisition and analysis. Populations were gated on lymphocyte, singlet, viable, CD3^+^, and CD8^−^ or CD4^+^ cells during flow cytometry analyses, unless otherwise specified in the figure legends.

### Polyamine assay

Polyamine content from cell lysates and culture supernatants were determined by fluorimetric method using a commercially available kit (BioVision #K475-100) as per manufacturer’s protocol. Briefly, cells from 4 wells (~4 × 10^6^) were harvested on day 4–7 post infection, and lysed in 100 μl of ice-cold polyamine lysis buffer using Dounce homogenizer with 15 firm strokes. The resulting homogenates were spun at 10,000 × *g* for 5 min at 4 °C. ~100 μl lysates were mixed with 2 μl of clean-up mix and incubated at room temperature for 30 min. Cell supernatants were similarly incubated with the cleanup mix. After the cleanup, the contents were transferred to a 10 kD MWCO filter (Thermo Pierce™ Protein Concentrators PES #88513) and spun at 10,000 × *g* for 20 min at 4 °C to remove high molecular weight proteins. The resulting filtrate was collected and stored at −80 °C until further analysis. A 10–20 μl aliquot of sample was dispensed into a 96 well black plate with flat bottom (BioVision M1355; Greiner Microlon plate) total volume was made up to 50 μl with polyamine assay buffer. To each well, 50 μl of the reaction mix containing polyamine enzyme, polyamine developer, and diluted polyamine probe were added. Simultaneously, respective blank wells were maintained by excluding the enzyme from the reaction mix and incubated at 37 °C for 30 min. The final product was read fluorometrically at 587 nm by exciting the samples at 535 nm using SpectraMax i3X microplate reader (Molecular Devices). Polyamine concentration was determined from the standard curve using the SoftMax Pro 6.1 software. For estimating the intracellular levels, polyamine concentrations were normalized to the viable cell numbers used to obtain assay lysates, given HIV induced cell death and reduced cell numbers in some cultures. The final results are expressed in pM/cell.

### Salivary metabolome analysis

Sample collection: Samples were collected in 50 mL sterile centrifuge tubes and immediately stored at −80 °C. 100 μl frozen aliquots of human saliva samples were processed for metabolite extraction liquid chromatography-mass spectrometry (LC-MS) analysis commercially by Creative Proteomics. Sample Preparation: 100 μl of samples were thawed, transferred to new tubes, extracted with 200 μl of 80% methanol, and vortexed for 30 s. Then the samples were kept at −40 °C for 1 h, vortexed for 30 s, and centrifuged at 10,000 × g at 4 °C for 15 min. Finally, 200 μl of supernatant and 5 μl of the internal standard DL-o-Chlorophenylalanine (1 mg/ml) were transferred to vial for LC-MS analysis. Quality control (QC) samples were used to evaluate the methodology. The same amount of extract was obtained from each sample and mixed as QC samples. The QC sample was prepared using the same sample preparation procedure.

Instrumental setup: Separation was performed by Ultimate 3000LC combined with Q Exactive MS (Thermo) and screened with ESI-MS (targeted MS/MS mode). The LC system is comprised of an ACQUITY UPLC HSS T3 (100 × 2.1 mm 1.8 μM) with Ultimate 3000LC. The mobile phase was composed of solvent A (0.05% formic acid-water) and solvent B (acetonitrile) with a gradient elution (0–1.0 min, 95% A; 1.0–12.0 min, 95%-5% A; 12.0–13.5 min, 5% A; 13.5–13.6 min, 5%–95% A; 13.6–16 min, 95% A). The flow rate of the mobile phase was 0.3 ml·min-1. The column temperature was maintained at 40 °C, and the sample manager temperature is set at 4 °C.

Mass spectrometry parameters in ESI+ and ESI− mode are listed as follows:

ESI+: Heater Temp 300 °C; Sheath Gas Flow rate, 45 arb; Aux Gas Flow Rate, 15 arb; Sweep Gas Flow Rate, 1 arb; spray voltage, 3.0 kV; Capillary Temp, 350 °C; S-Lens RF Level, 30%.

ESI-: Heater Temp 300 °C, Sheath Gas Flow rate, 45 arb; Aux Gas Flow Rate, 15arb; Sweep Gas Flow Rate, 1 arb; spray voltage, 3.2 kV; Capillary Temp,350 °C; S-Lens RF Level, 60%.

Bioinformatic data analysis included multivariate statistical analysis, single variable analysis, cluster analysis, and correlation network of differential metabolites. Statistically significant metabolites (FC > 1.5) were integrated with differentially expressed genes obtained from the RNAseq data and the combined data was visualized with Metaboanalyst (https://www.metaboanalyst.ca/) and Cytoscape v3.8 via Metscape v3.1 plugin (https://cytoscape.org/).

### RNA sequencing and metabolome data analysis

Gingival cells enriched in immune cells were prepared by removing epithelial cells based on gradient centrifugation. Human oral intraepithelial and lamina propria leukocytes (HOIL) samples from three control individuals were pooled and compared with three independent HIV+ individuals. RNA preparation, sequencing, and alignment were performed (Novogene)^4^. Briefly, NEB Next® Ultra™ RNA Library Prep Kit was used for strand-specific whole transcriptome sequencing library preparation. HiSeq2500 with Illumina TruSeq V4 chemistry (Illumina, CA) was used for sequencing the indexed RNA-seq libraries (HTSeq v0.6.1). The FASTQ files with 125 bp paired-end reads were processed using Trimmomatic (version 0.30) to remove adaptor sequences. The trimmed FASTQ data were aligned to the human genome with STAR (version 2.5), which used GENCODE gtf file version 4 (Ensembl 78). The gene reads count data from three independent human individuals were normalized with R Package limma (version 3.26.8) and analyzed with unpaired *t*-tests. Significantly up and downregulated genes in PLWH were analyzed in conjunction with metabolome analyses.

### Targeted polyamine quantification using LC-MS

#### Sample preparation

Standard solution: Serially diluted standard solutions containing standard substances of the 5 polyamines was prepared in 10% acetonitrile.

Sample solution: Cellular lysates were from three independent experiments were processed and run separately. 40 μL of the supernatant of each sample, once thawed on ice, or each of the standard solutions, was added with and 80 μL of 20 mM of dansyl chloride solution and 20 μL of borate buffer. The mixtures were reacted at 40 °C for 30 min.

#### LC-MS analysis

UPLC-MRM/MS analysis was performed using a commercial service facility at Creative Proteomics.10 μL aliquots of the resultant solutions were injected into a C18 LC column (2.1 × 150 mm, 1.8 μm) to run UPLC-MRM/MS on an Agilent 1290 UHPLC system coupled to an Agilent 6495B QQQ mass spectrometer operated in the positive-ion mode, with the use of 0.1% for mica acid in water (A) and acetonitrile (B) for binary gradient elution (50% to 100% B in 15 min), at 0.35 mL/min and 55 °C. The resulting data are normalized to input cells and expressed in per cell basis.

### Statistical analyses

*P < 0.05 was considered significant. Prism 8 (GraphPad Software, Inc.) was used to calculate *P* values. For random distribution, Mann–Whitney tests and for multiple comparisons between two or more groups one- or two-way ANOVA were used. Bonferroni *t*-test was the post hoc test used for multiple comparisons. An alpha value of *<0.05 was considered significant in correlation analysis, and spearman (r), and simple linear regression (R^2^) were used.

### Reporting summary

Further information on research design is available in the Nature Portfolio Reporting Summary linked to this article.

## Supplementary information

Supplementary Information

Reporting Summary

## Data Availability

The RNA sequencing data of controls generated in this study have been deposited in the GEO, NCBI database under accession code GSE167211. The RNA sequencing data of PLWH generated in this study have been deposited in the NCBI Genotypes and Phenotypes (dbGaP) data repository under accession code dbGaP Study Accession: phs002364.v1.p1. The metabolome LC/MS data as well as the processed metabolic profiles and corresponding metadata for the human (deidentified) samples have been deposited in the Metabolomics Workbench repository under accession code NMRD: ST002328. Source data are provided with this paper.

## Source data

Source Data
