# Supplementary material for: Polyamine metabolism impacts T cell dysfunction in the oral mucosa of people living with HIV
Source: Nat Commun. 2023 Jan 25;14:399. doi: 10.1038/s41467-023-36163-2 (PMC9873639; doi:10.1038/s41467-023-36163-2)
Supplement: Supplementary file 1 — Supplementary Information [file 41467_2023_36163_MOESM1_ESM.pdf]

Supplementary Figures and Table

Polyamine metabolism impacts T cell dysfunction in the oral mucosa of people living with HIV

Supplementary Fig. 1

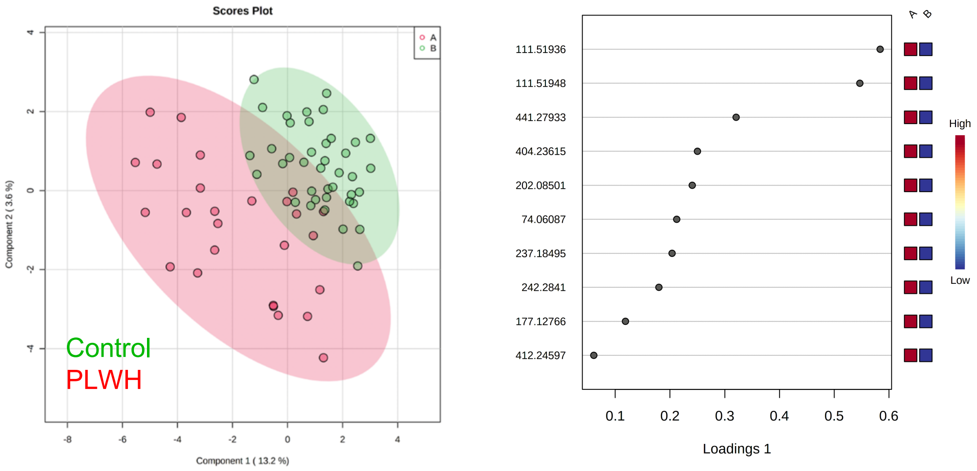

Supplementary Fig.1. PLS-DA plot of salivary metabolomics data. Saliva samples were collected. (uninfected controls n= 26; PLWH; n= 40). PLS-DA plot was created using raw data and metabolites were identified via m/z value as described in methods (left). Variable importance in projection (VIP) score plot for the top 10 most important metabolite features identified by PLS-DA (right).

Supplementary Fig. 2

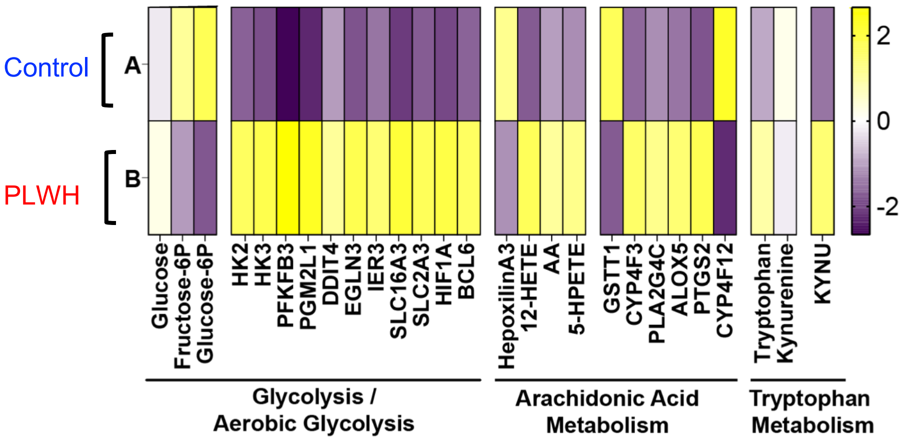

Supplementary Fig.2. Heatmap of log2 fold changes in metabolites and related genes involved in glycolysis, arachidonic acid metabolism, and tryptophan degradation. FC cutoff is 1.15 or Log2FC=.2 for metabolites. FC cutoff for genes is 2, or Log2FC=1.

Supplementary Fig. 3

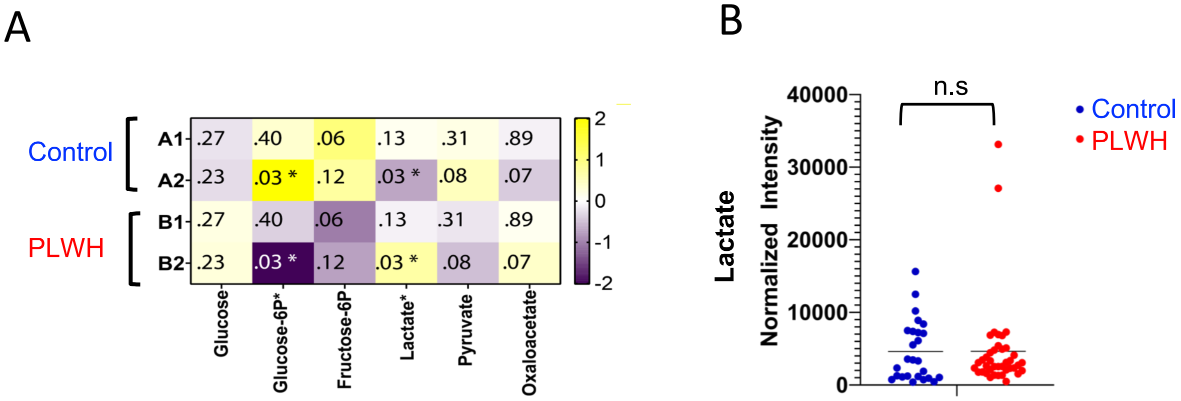

Supplementary Fig.3. Salivary metabolite comparison between control and PLWH groups (uninfected controls n= 26; PLWH; n= 40). **A)**. Heatmap of Log2FC of metabolite intensity for glycolytic and TCA pathway metabolites in age-based subgroups. P-values for each comparison are provided. FC cutoff is 1.1 (Log2FC .1). **B**) Scatterplot of lactate normalized intensity. Unpaired, two-tailed, student t-test with Welch's correction. P= 0.98; non-significant.

Supplementary Fig. 4

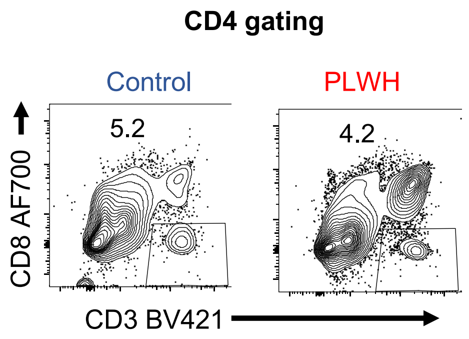

Supplementary Fig.4. Gating strategy used for the flow cytometry analysis exemplifying the gates used for $CD4^+$ cell gating in figures 1 - 9. HOIL were processed for flow cytometry *ex vivo* or after PMA/Ionomycin re-stimulation. $CD4^+$ T cells were gated by either gating on CD8 negative $CD3^+$ cells, or $CD4^+CD3^+$ cells. These were pre-gated on FSC/SCC, viable cells, and singlets.

Supplementary Fig. 5

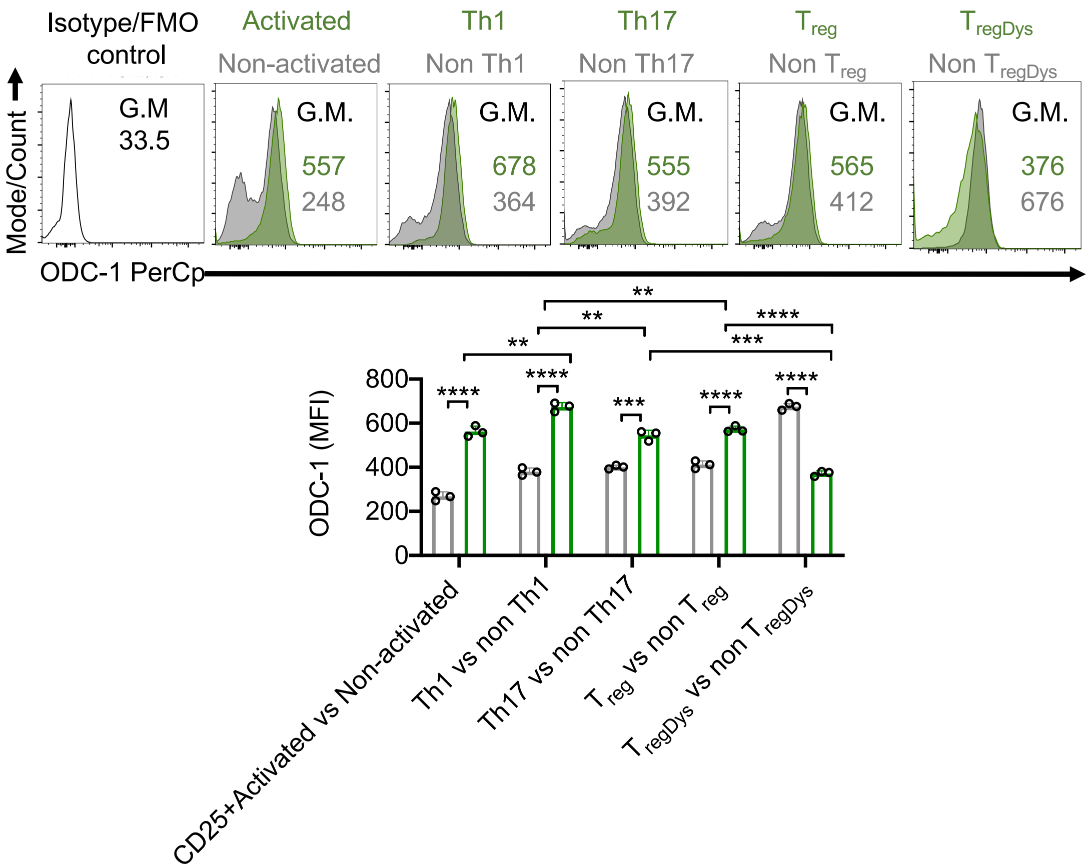

Supplementary Fig.5. **ODC-1 expression in different CD4$^+$T cells subsets. T_{regDys} subset shows the least ODC-1 expression.** HTOC were activated by TCR stimulation. Flow cytometry was performed to determine ODC-1 expression in different CD4$^+$ T cell subsets in cultures, 6 days post-stimulation. Representative flow cytometric data showing results independent tonsil donors (n=3) (above). Statistical quantification of ODC-1 expression (below), showing Mean +/- SEM (n=3). Multiple T tests- Two-tailed; One unpaired t test per row. **** P> 0.00001 < 0.00009; *** P> 0.0002 < 0.0005; **P < 0.003.

Supplementary Fig. 6

A

	T_{reg} cell number	T_{reg}Dys cell number	Th17 cell number
Uninfected	8150	910	8305
HIV	3648	601	561
Uninfected + ARI	8649	808	8285
HIV+ ARI	6935	1194	3604

B

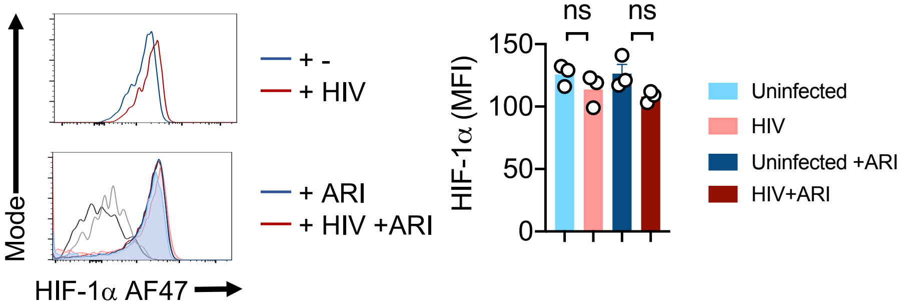

Supplementary Fig.6. HTOC were activated by TCR stimulation, infected with HIV on day 1 after TCR stimulation and allowed to expand with ARI. Flow cytometry was performed to determine the cell numbers of T_{reg}, T_{regDys}, and Th17 subsets as in Figure 2. **A)** Cell numbers of Th subsets in HTOC cultures are shown. **B) HIV-1 does not regulate HIF-1α in CD4+ T cells.** HIF-1α expression was determined in CD4$^+$ T cells, 6 days post-infection (left). Representative flow cytometric data quantification (right) from three independent tonsil donors. ns= non significant. Mean values +/- SEM; Two-tailed; Unpaired t test.

Supplementary Fig. 7

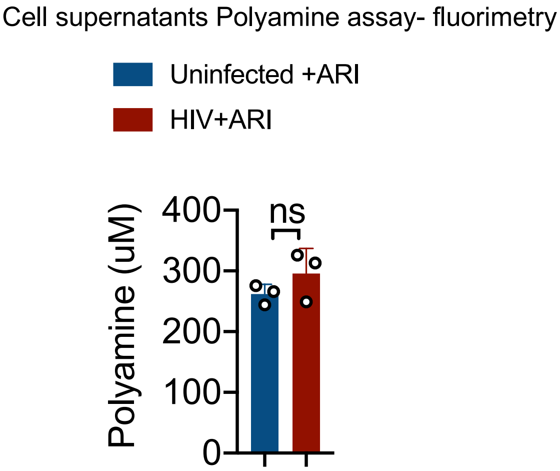

Supplementary Fig.7. HIV does not increase polyamines in the supernatants. HTOC were TCR activated, infected with HIV, and allowed to expand with ARI. Fluorimetric estimation of polyamines in cell culture supernatants. Collated data from day 4 and day 7 post-infection supernatants are presented. ns= non significant; Unpaired t test with Welch's correction; Two-tailed; n=3 independent experiments; Mean values +/- SD.

Supplementary Fig. 8

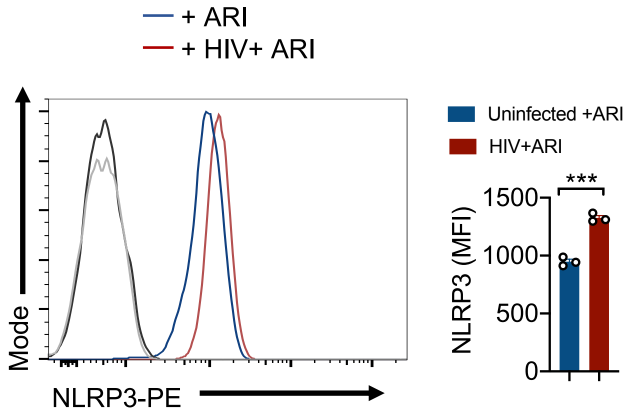

Supplementary Fig.8. HIV-1 upregulates NLRP3. HTOC were activated by TCR stimulation, infected with HIV on day 1 after TCR stimulation and allowed to expand with ARI. Flow cytometry was performed to determine NLRP3 expression in $CD4^+$ T cells, 6 days post-infection. Representative flow cytometric data (left) and statistical quantification (right) from three independent tonsil donors. Light and dark grey histograms represent staining controls for ARI and HIV+ARI conditions respectively. Mean values +/- SEM; *** P = 0.0002; Two-tailed; Unpaired t test.

Supplementary Fig. 9

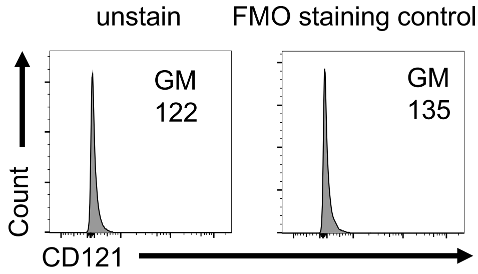

Supplementary Fig.9. CD121 staining controls. HTOC $CD4^+$ T cells were TCR activated with no HIV infection. Flow cytometry histograms of unstain and FMO controls for CD121staining are shown.

Supplementary Fig. 10

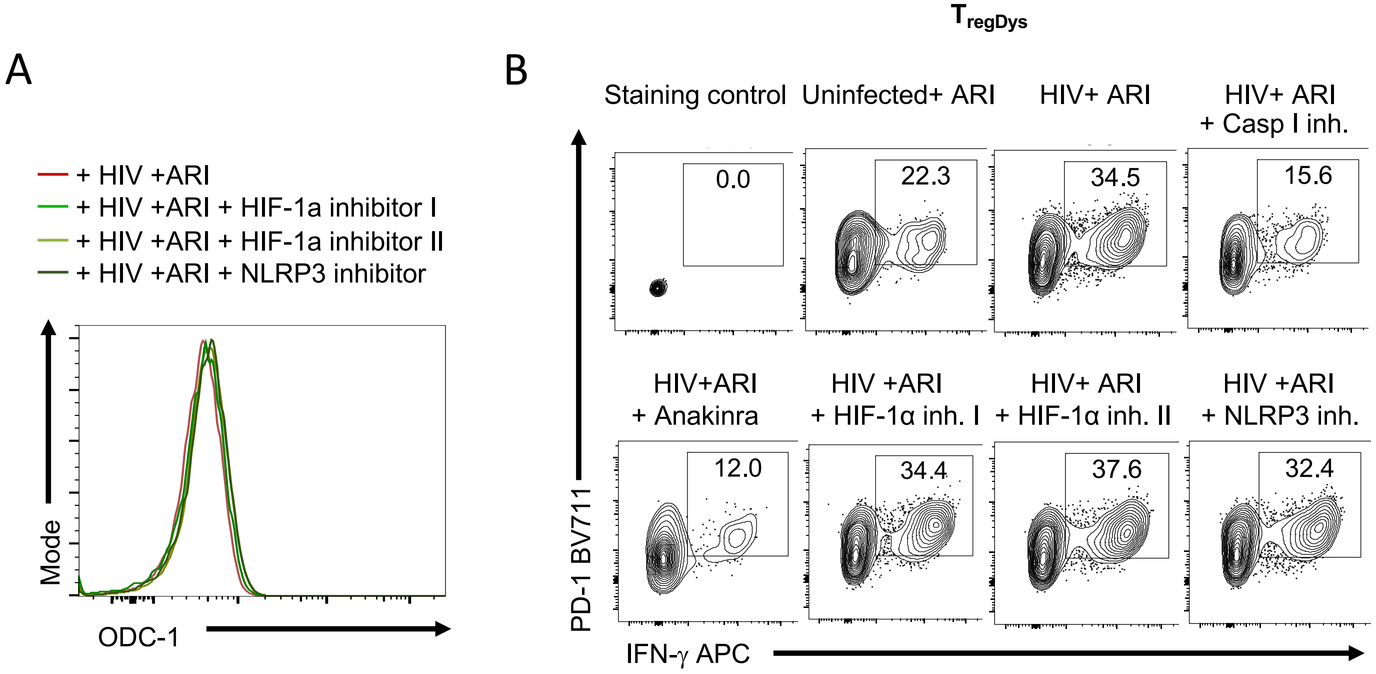

Supplementary Fig.10. **Neither NLRP3 nor HIF-1α regulates ODC-1 expression and T_{regDys} induction during HIV infection in $CD4^+$ T cells.** HTOC were activated by TCR stimulation, infected with HIV on day 1 after TCR stimulation and allowed to expand with ARI. HIF-1α inhibitor I(Echinomycin; 5 nM) , HIF-1α inhibitor II (Oridonin; 1 μg/ml)), NLRP3 inhibitor (MCC950; 20 μM), Caspase-1 inhibitor (VX-765, 250nM), and Anakinra (10 μg/ml) were added as indicated, 36 hours post infection. Flow cytometry was performed to determine ODC-1 expression (A) and the frequency of PD-1^+IFN-γ^+FOXP3^+T_{regDys} cells (B) in $CD4^+$ T cells, 6 days post-infection. Representative flow cytometric data from one of three independent experiments. FMO staining control is shown in the first panel in B.

Supplementary Fig. 11

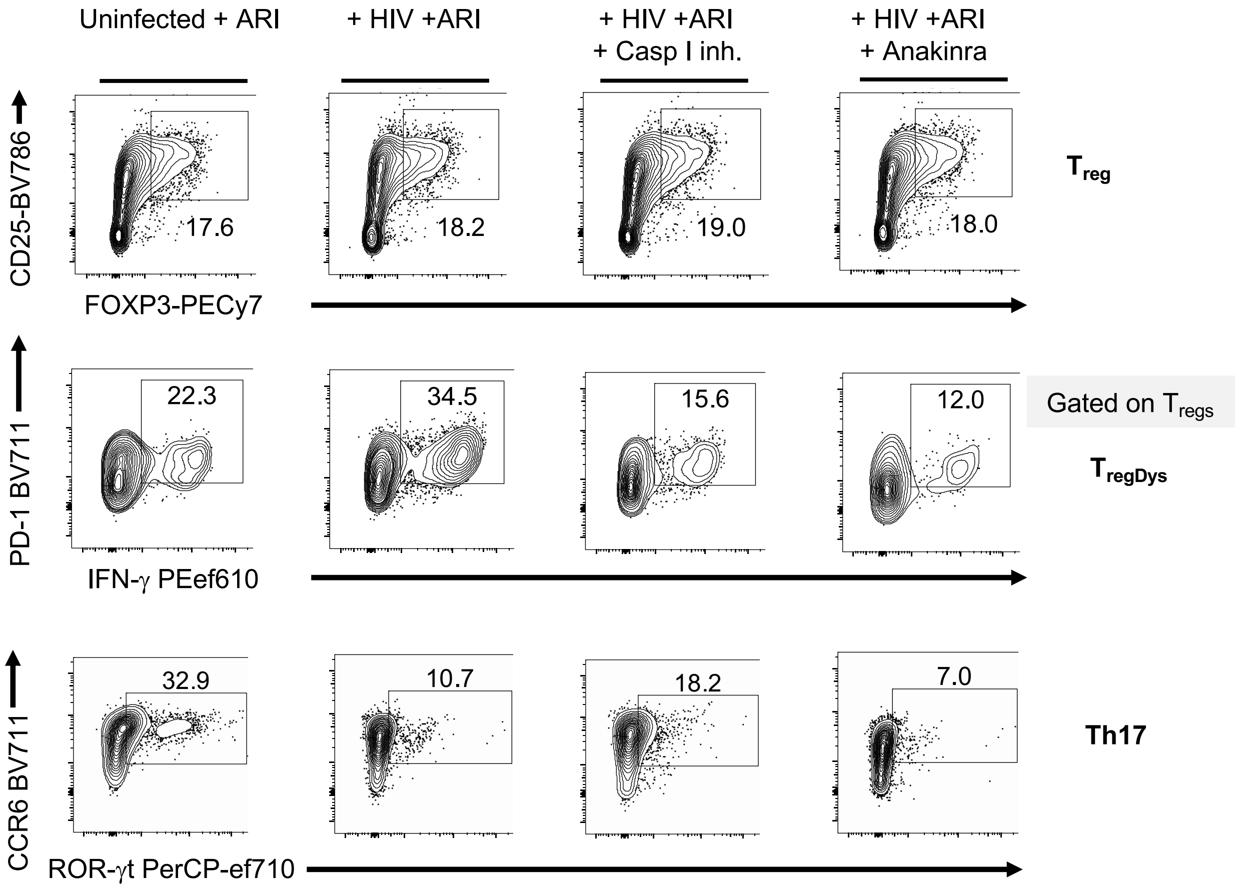

Supplementary Fig.11. **Effects of Caspase and IL-1β inhibition on T_{regs}, T_{regDys}, and Th17 cells.** HTOC were activated by TCR stimulation, infected with HIV on day 1 after TCR stimulation. ARI, Caspase-1 inhibitor (VX-765, 250nM), and Anakinra (10 μg/ml) were added as indicated, 36 hours post infection. Flow cytometry was performed to determine the proportions of T_{regs} (top), T_{regDys} (middle) and $CCR6^{+}$ROR-γt^{+}Th17 cells (bottom) in $CD4^{+}$ T cells, 6 days post-infection.

Supplementary Fig. 12

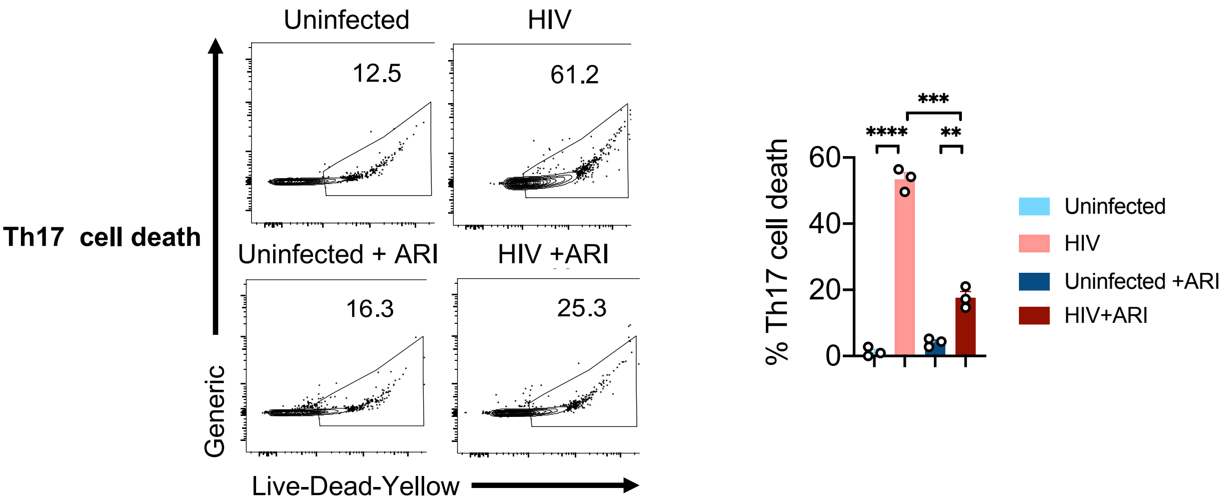

Supplementary Fig.12. Th17 cell loss is due to cell death during HIV infection in CD4+ T cells, which is partially restored by ARI. HTOC were activated by TCR stimulation, infected with HIV on day 1 after TCR stimulation. ARI was added to some cultures 36 hr after infection. Flow cytometry was performed to determine cell death using live-dead staining (pre-gated on CCR6+ROR-γt+Th17 cells) in CD4+ T cells, 6 days post-infection. Representative flow cytometric data (left) from three independent tonsil donors and are presented as mean values +/- SEM (right). **** P< 0.0001; *** P< 0.0001; **P <0.02; Two-tailed; Unpaired t test.

Supplementary Fig.13

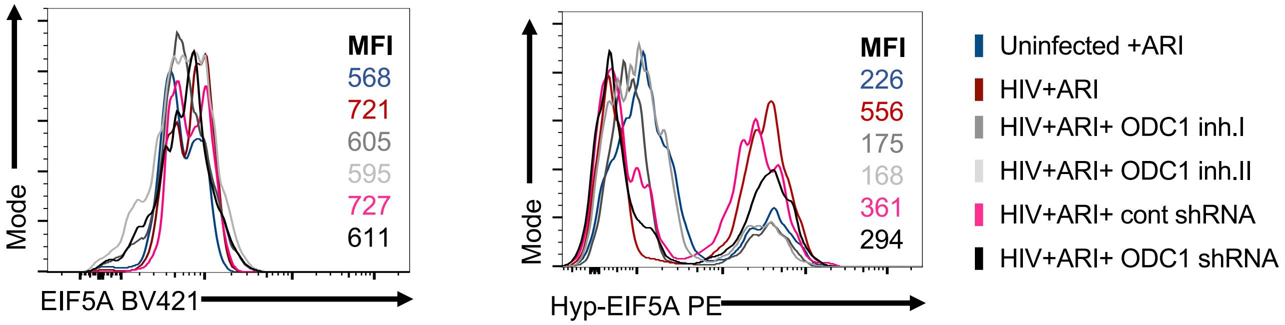

Supplementary Fig.13 ODC-1 activity upregulates EIF5A and hypusinated-EIF5A levels during HIV infection. Tonsil CD4+ T cells were TCR activated, infected with HIV and allowed to expand with ARI. DFMO (ODC-1 inhibitor I; 2.5 mM), POB (ODC-1 inhibitor II; 100 μM), Control shRNA lentiviral particles-A with polybrene (2 μg/ml), and ODC1 shRNA lentiviral particles with polybrene (2 μg/ml) were added as indicated, 36 hours post infection. Flow cytometry was performed to determine the proportions of EIF5A expression(left) and hypusinated EIF5A expression (right) in CD4+ T cells, 6 days post-infection.

Supplementary Fig. 14

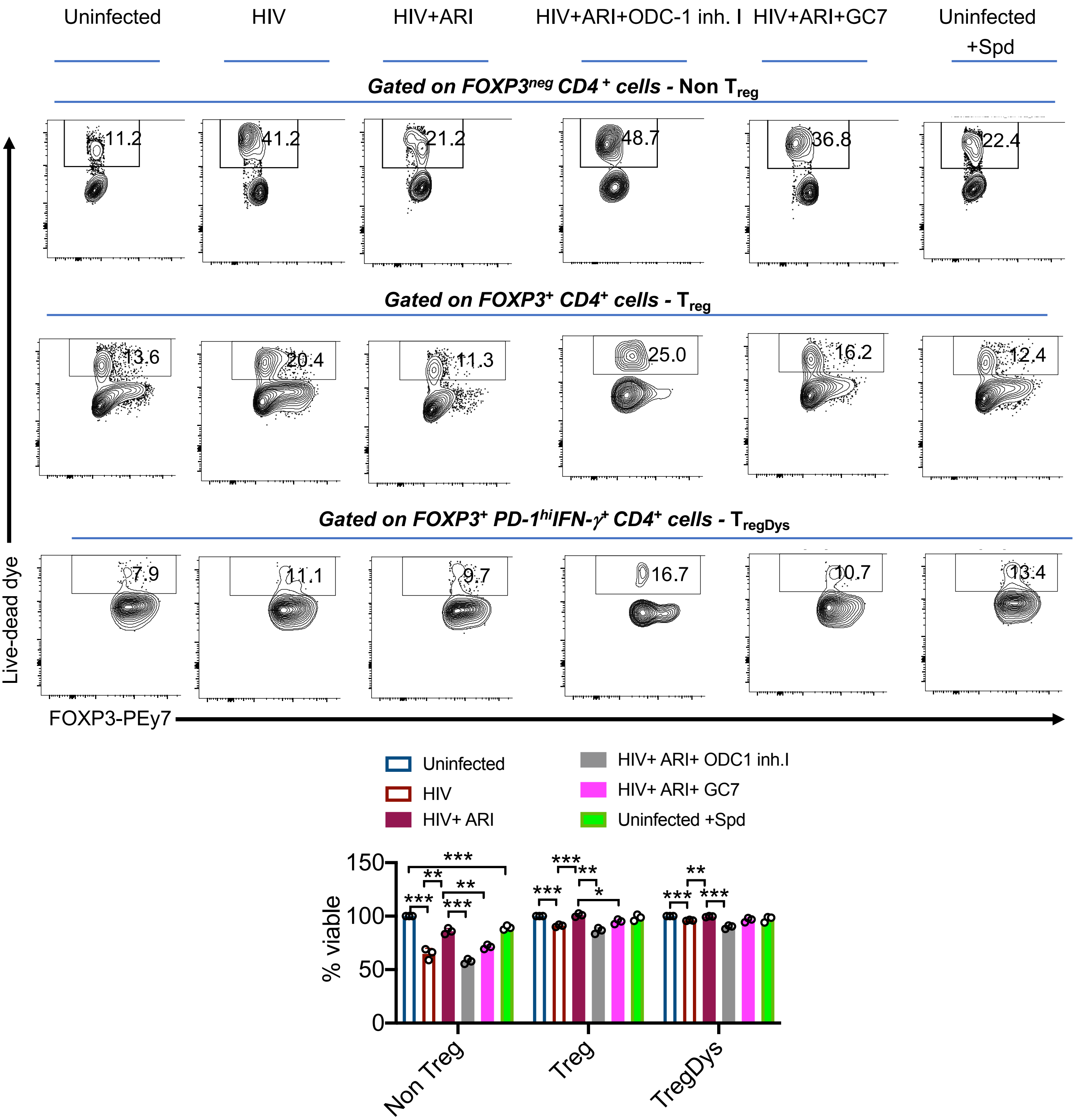

Supplementary Fig.14. ODC-1 inhibition and GC7 reduce CD4 viability and the effect is more pronounced in FOXP3^neg T cells. HTOC CD4⁺ T cells were stimulated as described in methods. Some cultures were HIV-infected with or without ARI, ODC-1 inhi I., or GC7. Other uninfected cultures were treated with Spermidine. Viability was determined by live-dead flow cytometry staining on day 6 after stimulation. Contour plot gates show the % dead cells (above). Representative flow cytometric data from one of at least three independent tonsil donors. Statistical quantification of % viability normalized to uninfected controls (below), showing three mean values of the triplicate experiments. Multiple T tests- Two-tailed; One unpaired t test per row. *** P> 0.0001 < 0.0009; ** P> 0.001 < 0.003; *P = 0.0183.

Supplementary Fig. 15

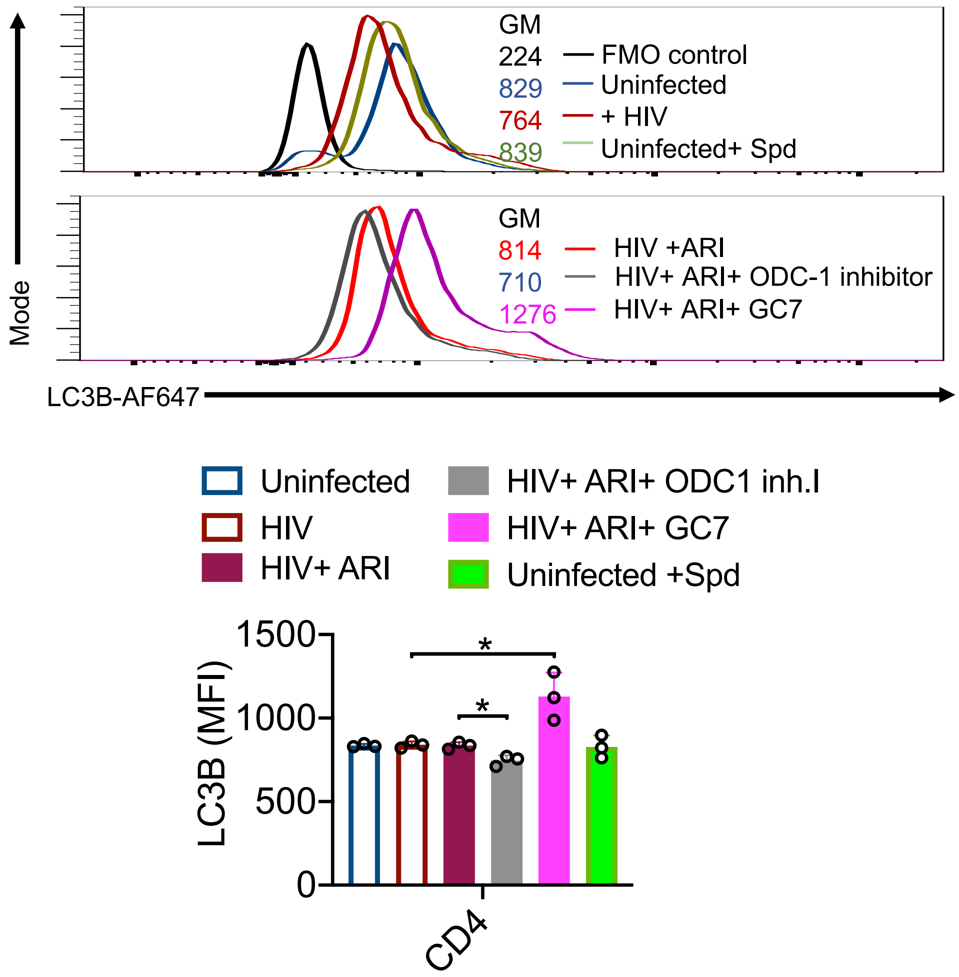

Supplementary Fig.15. Reduced CD4 viability by ODC-1 inhibition does not involve the upregulation of autophagic protein LC3B. GC7 increases LC3B. HTOC $CD4^{+}$ T cells were stimulated as described in methods. Some cultures were HIV-infected with or without ARI, ODC-1 inhi I., or GC7. Other uninfected cultures were treated with Spermidine. LC3B expression was determined by intracellular flow cytometry staining on day 6 after stimulation. Histograms show respective geometric mean (G.M) fluorescence intensity (MFI) of LC3B staining (above). Representative flow cytometric data from one of at least three independent tonsil donors. Statistical quantification of MFI (below), showing three mean values of the triplicate experiments. Multiple T tests- Two-tailed; One unpaired t test per row. *P =0.0149.

Supplementary Fig. 16

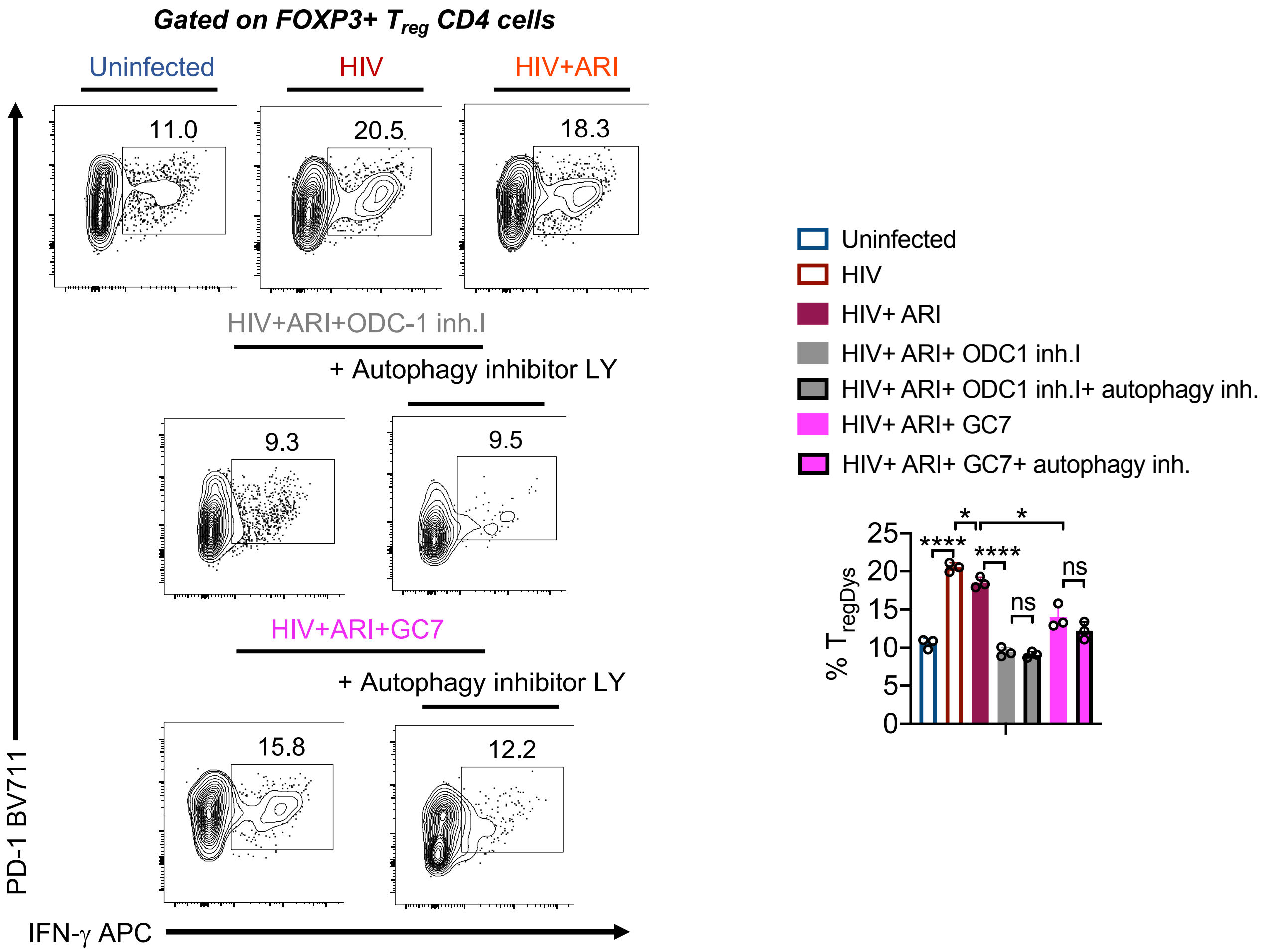

Supplementary Fig.16. Reduced T_{regDys} caused by ODC-1 inhibition or GC7 does not involve autophagic cell death. HTOC CD4+ T cells were stimulated as described in methods. Some cultures were HIV-infected with or without ARI, ODC-1 inhi I., or GC7 in the presence or absence of LY294002 (10 uM). Other uninfected cultures were treated with Spermidine. % T_{regDys} was determined by intracellular flow cytometry staining on day 6 after stimulation. Contour plot gates show the % T_{regDys} (left). Representative flow cytometric data from one of at least three independent tonsil donors. Statistical quantification of % T_{regDys}(right). n=3 independent experiments. Mean values +/- SD. Multiple T tests- Two-tailed; One unpaired t test per row. **** P> 0.00004 < 0.00007; * P > 0.0107 < 0.0209.

Supplementary Fig. 17

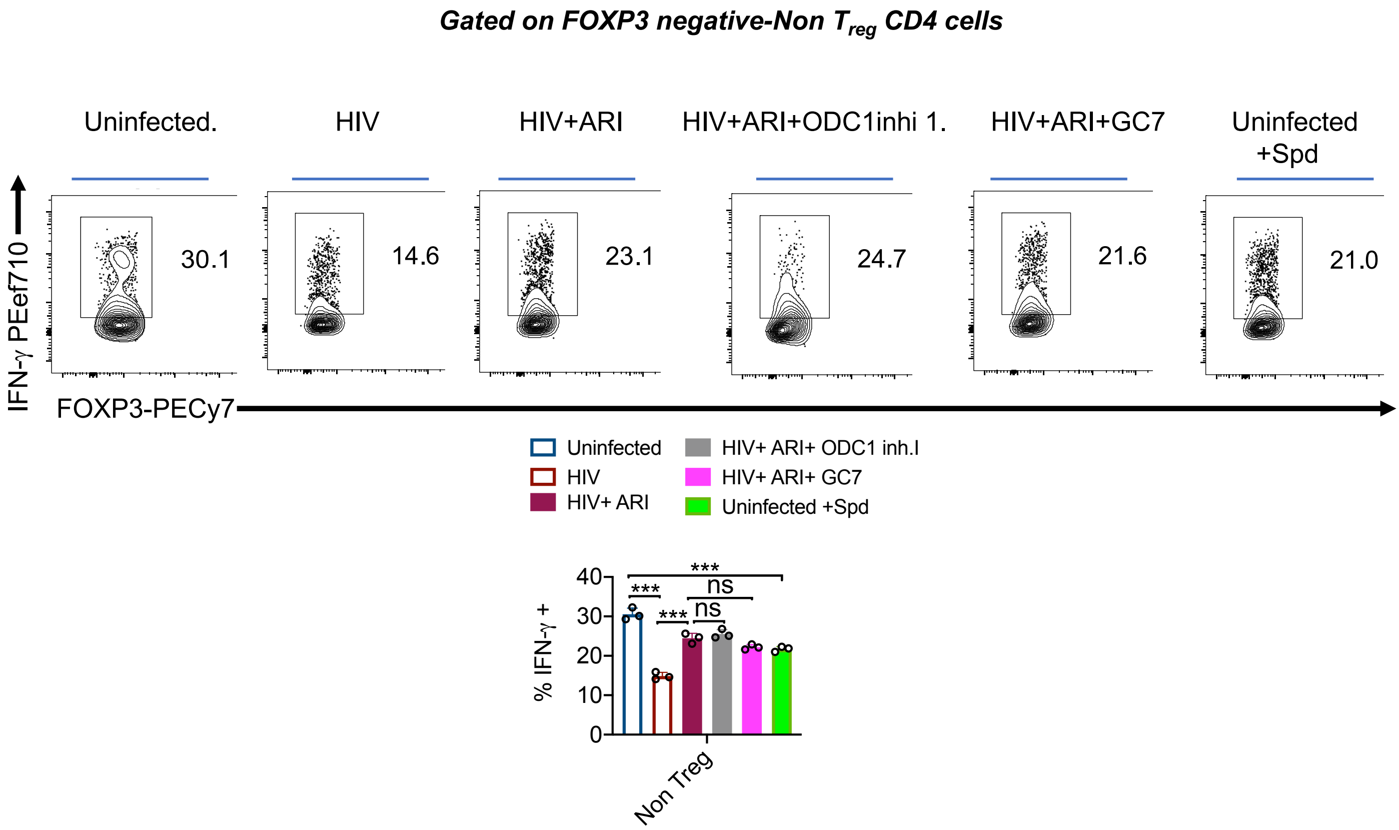

Supplementary Fig.17. **HIV infection and exogenous addition of polyamines do not increase but reduce IFN-γ⁺ among non T_{regs}. ODC-1 inhibition of EIF5A hypusination inhibition do not affect IFN-γ⁺ cells in HIV-infected cultures.** Tonsil CD4⁺ T cells were TCR stimulated in the presence of GC7 (10 μM), Putrescine dihydrochloride (100 μM), or Spermidine (1mM) with no infection. Contour plots (above) and statistical quantification of % IFN-γ⁺ cells based on the flow cytometry analysis on day 6. n=3 independent experiments. Mean values +/- SD. Multiple T tests- Two-tailed; One unpaired t test per row. *** P> 0.0001 < 0.0008; ns= non significant.

Supplementary Fig. 18

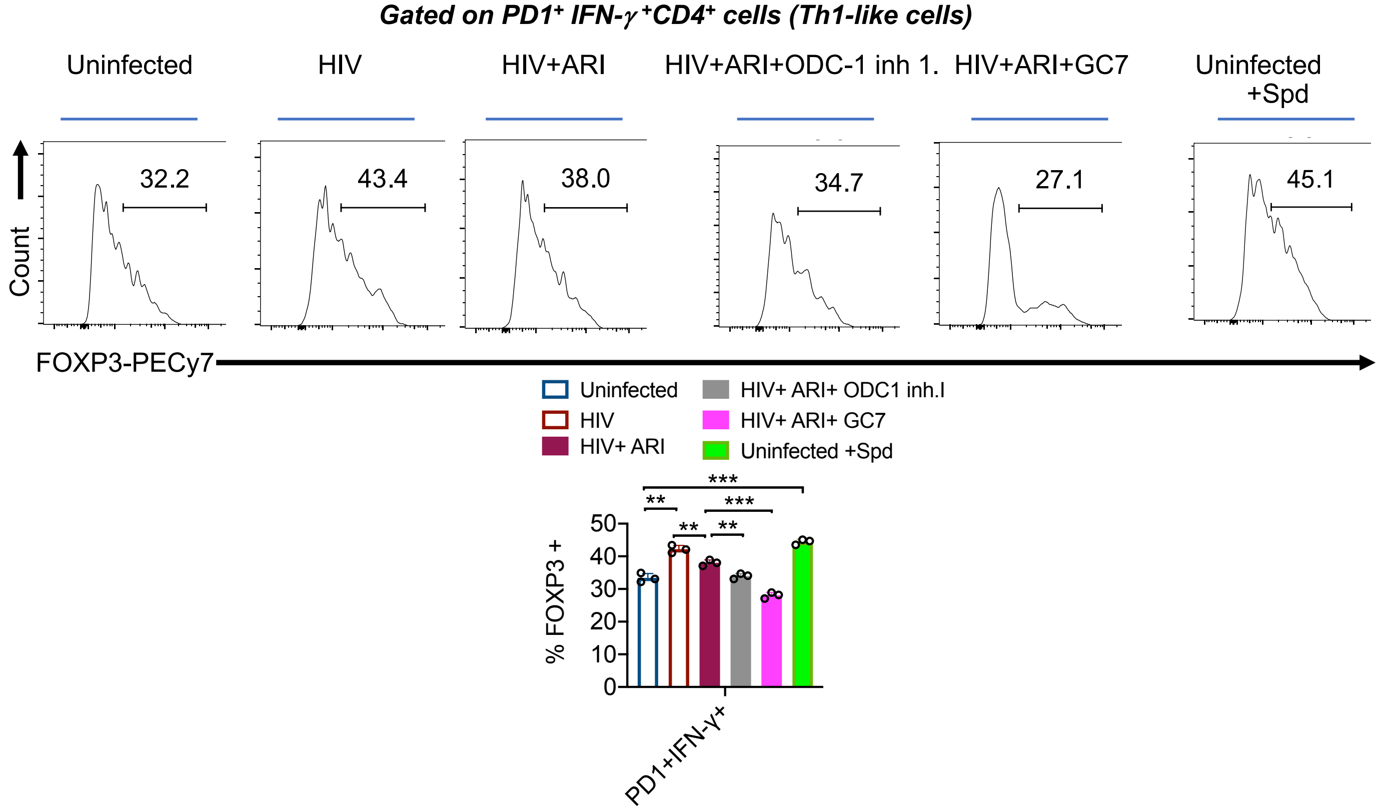

Supplementary Fig.18. HIV infection and exogenous addition of polyamines induce FOXP3 in PD-1+ IFN-γ+ CD4+cells. Reduced T_{regDys} caused by ODC-1 inhibition or GC7 involves blockade of FOXP3 induction in PD-1+ IFN-γ+ CD4+cells during HIV-infection. HTOC CD4+ T cells were stimulated as described in methods. Some cultures were HIV-infected with or without ARI, ODC-1 inhi I., or GC7. Other uninfected cultures were treated with Spermidine. FOXP3 expression was determined in PD1+ IFN-γ +CD4+ cells by intracellular flow cytometry staining on day 6 after stimulation. Contour plot gates show the % FOXP3+ cells (above). Representative flow cytometric data from one of at least three independent tonsil donors. Statistical quantification of % FOXP3 (below); n=3 independent experiments. Mean values +/- SD. Multiple T tests- Two-tailed; One unpaired t test per row. ** P=0.001-0.004; *** P= 0.0001-0.0002.

Supplementary Fig. 19

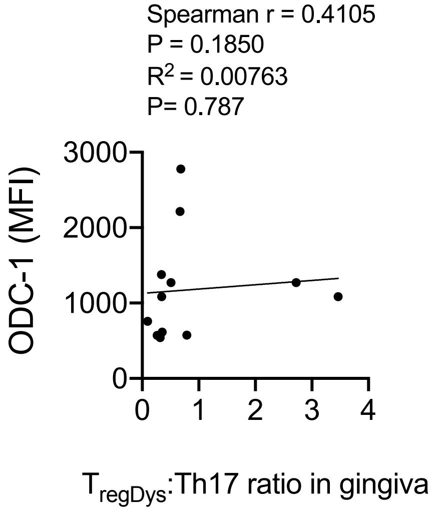

Supplementary Fig.19. ODC-1 expression does not correlate with T_{reg}/Th17 ratio in oral gingival CD4+ T cells. Gingival oral mucosal cells from healthy controls (n=19) and PLWH (n=30) were processed for flow cytometry ex vivo to determine % T_{regDys}, %T_{reg} and ODC-1 expression. Correlation plot showing the absence of correlation between gingival T_{regDys} /Th17 ratios and ODC-1 expression in CD4+ T cells (n = 21). P values from both correlation and regression analysis are provided.

Supplementary Fig.20

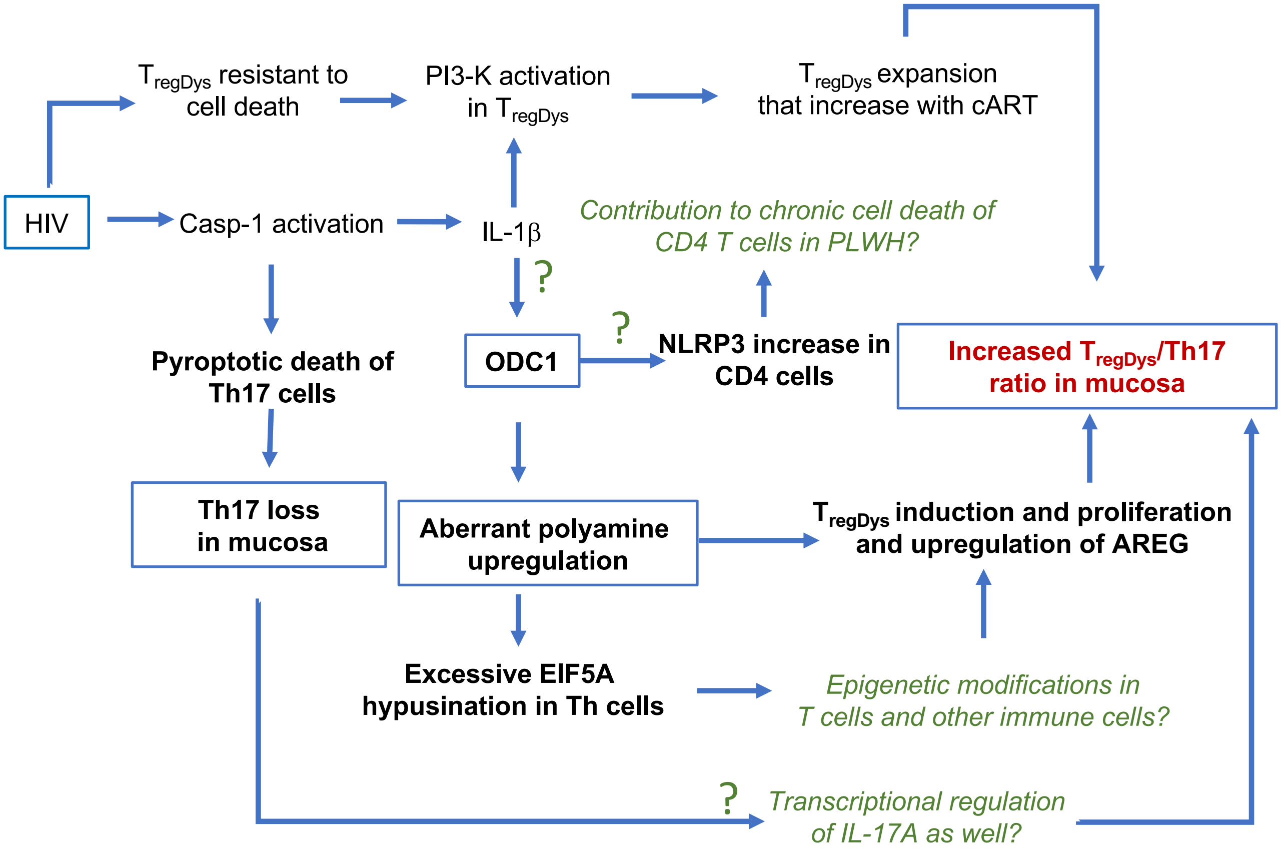

Supplementary Fig.20. Graphical abstract. HIV mediated polyamine synthesis and Th17 loss are critical for causing Th dysregulation. Aberrantly increased caspase-1, IL-1β, ODC-1 expression, and polyamine synthesis drive Th dysregulation and the titling of Th17/T_{regDys} ratios. The mechanism by which IL-1β increases ODC-1 expression remains to be studied. Also, the role of possible epigenetic modifications in cytokine genes due to hyper EIF5A hypusination in $CD4^+$ T cells and other immune cells will be addressed in future studies.

Supplementary Table 1

Group	Control (n = 32)	PLWH (n=46)
**Age (years) median**	49 +/- 18.5	54 +/- 9.9
**Minimal age**	18	18
**Aged 60 and above**	21.8%	22.2%
**Time under cART median**	NA	15 +/- 7.6 yrs
**Viral load median**	NA	20 (range 0.8 – 272)
**% prior Candidiasis positive**	0	34.7%
**% periodontitis +**	0	4.3%
**Inclusion criteria**	Age-18 years and above #	Age-18 years and above HIV positive with cART treatment for at least 1 year #.
**Exclusion criteria**	Oral inflammatory lesions (including gingivitis and periodontitis), oral cancer diagnosis, soft tissue lesions, and the use of tobacco in the past month.	Oral cancer diagnosis and the use of tobacco in the past month*

$CD4^+$ counts were at least 350 - 700/μl for the control and PLWH patients.
*The absence of tobacco use was confirmed by Cotinine ELISA in saliva.
For the metabolome analysis, randomly chosen saliva samples that were available in sufficient quantities were used; Uninfected controls (Group A; n= 26) and PLWH (Group B; n= 40).Similarly, flow cytometry results are based on the data from a subset of randomly chosen samples within the groups (See reference).

Reference

Bhaskaran, N. et al. Oral immune dysfunction is associated with the expansion of FOXP3+PD-1+Amphiregulin+ T cells during HIV infection. Nature Communications 12, 5143 (2021).
