# Supplementary material for: Polyamine metabolism impacts T cell dysfunction in the oral mucosa of people living with HIV
Source: Nat Commun. 2023 Jan 25;14:399. doi: 10.1038/s41467-023-36163-2 (PMC9873639; doi:10.1038/s41467-023-36163-2)
Supplement: Supplementary file 2 — Reporting Summary [file 41467_2023_36163_MOESM2_ESM.pdf]

nature portfolio

Corresponding author(s): Pushpa Pandiyan

Last updated by author(s): Jan 9, 2023

Reporting Summary

Nature Portfolio wishes to improve the reproducibility of the work that we publish. This form provides structure for consistency and transparency in reporting. For further information on Nature Portfolio policies, see our Editorial Policies and the Editorial Policy Checklist.

Statistics

For all statistical analyses, confirm that the following items are present in the figure legend, table legend, main text, or Methods section.

n/a	Confirmed	
☐	☒	The exact sample size (n) for each experimental group/condition, given as a discrete number and unit of measurement
☐	☒	A statement on whether measurements were taken from distinct samples or whether the same sample was measured repeatedly
☐	☒	The statistical test(s) used AND whether they are one- or two-sided<br>*Only common tests should be described solely by name; describe more complex techniques in the Methods section.*
☐	☒	A description of all covariates tested
☐	☒	A description of any assumptions or corrections, such as tests of normality and adjustment for multiple comparisons
☐	☒	A full description of the statistical parameters including central tendency (e.g. means) or other basic estimates (e.g. regression coefficient) AND variation (e.g. standard deviation) or associated estimates of uncertainty (e.g. confidence intervals)
☐	☒	For null hypothesis testing, the test statistic (e.g. F, t, r) with confidence intervals, effect sizes, degrees of freedom and P value noted<br>*Give P values as exact values whenever suitable.*
☒	☐	For Bayesian analysis, information on the choice of priors and Markov chain Monte Carlo settings
☒	☐	For hierarchical and complex designs, identification of the appropriate level for tests and full reporting of outcomes
☐	☒	Estimates of effect sizes (e.g. Cohen's d, Pearson's r), indicating how they were calculated

Our web collection on statistics for biologists contains articles on many of the points above.

Software and code

Policy information about availability of computer code

Data collection	For Flow cytometry: Becton Dickinson Fortessa -BD FACSDiva software ver.7<br>For RNA seq: Illumina Casava1.7 software used for basecaling. HTSeq v0.6.1; STAR v2.5; Trimmomatic (version 0.30) (to remove adaptor sequences), and GENCODE gtf file version 4 (Ensembl 78) to generate gene reads count data.<br><br>For Metabolome :<br>Metabolite identifications were made with Compound Discoverer 3.1 SP1 and MZmine2 software. MS1 and MS2 spectra were matched using a mass tolerance of m/z =0.1. The raw data were acquired and aligned using the Compound Discover based on the m/z value and the retention time of ion signals. Ions from both ESI- or ESI+ were merged and imported into the SIMCA-P program (version 14.1) for multivariate analysis. Further data processing was performed by using DecoID (DecoID v0.3.0) to deconvolute chimeric MS2 spectra and increase the identification rate. Metabolite identifications were made with level 2 confidence according to the Metabolomics Standards Initiative.<br><br>For polyamine concentration: SoftMax Pro 6.1 software
Data analysis	Flowjo versions 9.8, 9.9.6, 10.5.3, and 10.7.1<br>Graph Pad ver Prism 8<br>Microsoft Excel 2016, ver 16.16.27<br>RNA sequencing differential Analysis using Deseq 2,v2_1.6.3, ClusterProfiler v2.4.3, and EdgeR (version 3.26.8);<br>Heatmaps in R using the heatmap 2 functioning plots (version 2.17.0)<br>RNA sequencing Enrichment Analysis using GSEA (http://www.broad.mit.edu/GSEA), Molecular Signatures Database v7.4 (http://www.gsea-msigdb.org/gsea/msigdb/index.jsp)

Differential gene expression analysis: The gene reads count data from HOIL and PBMC samples were normalized with Edge R Package limma (version 3.26.8) and analyzed with an unpaired t-test.
Reactome Pathway Database (https://reactome.org), and Gene Ontology enrichment analysis (http:// geneontology.org) databases
Metabolome analysis: Metaboanalyst 5.0. Graphs were generated via Metascape

For manuscripts utilizing custom algorithms or software that are central to the research but not yet described in published literature, software must be made available to editors and reviewers. We strongly encourage code deposition in a community repository (e.g. GitHub). See the Nature Portfolio guidelines for submitting code & software for further information.

Data

Policy information about availability of data

All manuscripts must include a data availability statement. This statement should provide the following information, where applicable:

- Accession codes, unique identifiers, or web links for publicly available datasets
- A description of any restrictions on data availability
- For clinical datasets or third party data, please ensure that the statement adheres to our policy

RNA sequencing data have been deposited in GEO, NCBI with the GSE167211 accession code. https://www.ncbi.nlm.nih.gov/geo/query/acc.cgi?acc=GSE167211 and at the NCBI Genotypes and Phenotypes (dbGaP) data repository (dbGaP Study Accession: phs002364.v1.p1) and available at https://www.ncbi.nlm.nih.gov/projects/gap/cgi-bin/study.cgi?study_id=phs002364.v1.p1.
Other source data will be provided as a source file with this manuscript.
The raw LC/MS data as well as the processed metabolic profiles and corresponding metadata for the human (deidentified) samples are publicly available on the Metabolomics Workbench repository (NMRD: ST002328).

Human research participants

Policy information about studies involving human research participants and Sex and Gender in Research.

Reporting on sex and gender: Participants of both sexes were recruited.

Population characteristics: Informed consent were obtained from healthy individuals (n = 32) and Cleveland HIV+ cohort (n = 46). Healthy control subjects were at least 18 years of age and in good general health. HIV+ participants were 18 years or older, and were HIV positive with cART treatment for at least 1 year. Characteristics of enrolled participants were provided in supplementary table 1.

Recruitment: Participants were recruited after obtaining informed consents. Healthy control subjects were at least 18 years of age and in good general health. Exclusion criteria were oral inflammatory lesions (including gingivitis and periodontitis), oral cancer diagnosis, soft tissue lesions, and the use of tobacco in the past month. HIV+ participants were 18 years or older, and were HIV positive with cART treatment for at least 1 year. Exclusion criteria were oral cancer diagnosis and the use of tobacco in the past month. The inclusion and exclusion criteria were the same for periodontitis study, except that the the inclusion criteria for the periodontitis group included the presence of periodontitis.
HIV+ individuals volunteered based on their HIV positivity. Studying the HIV+ patients in this Case-Control study was the goal of the investigation and this self-selection did not impact the results negatively.

Ethics oversight: University Hospitals Cleveland Medical Center Institutional Review Board (IRB); IRB# 05-17-02

Note that full information on the approval of the study protocol must also be provided in the manuscript.

Field-specific reporting

Please select the one below that is the best fit for your research. If you are not sure, read the appropriate sections before making your selection.

☒ Life sciences ☐ Behavioural & social sciences ☐ Ecological, evolutionary & environmental sciences

For a reference copy of the document with all sections, see nature.com/documents/nr-reporting-summary-flat.pdf

Life sciences study design

All studies must disclose on these points even when the disclosure is negative.

Sample size: Power analyses was done based on estimates. For the power and sample size calculation, we used https://www.stat.ubc.ca/~rollin/stats/ssize/n2.html We used relevant population values for mu1 (mean of population 1), mu2 (mean of population 2), and sigma value of 0.5 (common standard deviation) 0.05 as α (type I error rate) and a 2 sided test for power calculation. Because our preliminary data showed that HIV+ group had atleast 1.4 fold increase in Treg proportions, we used 1 and 1.4 as mu1 and mu2 respectively. Our desired power was set to 0.8.

Data exclusions: No data points were excluded

Replication: We performed at least triplicate repeats of the in vitro experiments with independent biological replicates in each experiment. We confirm

Replication	that all replicate experiments showed reproducibly similar data and were successful except for a 2-3 episodes of contamination in which cases data were not collected. For ex vivo analyses of human samples, samples from each patient were used as biologically independent replicates.
Randomization	In vitro experiments were also performed using randomly allocated tonsils.Human participants were randomly recruited for control and HIV+ groups, with representation of males and females in each group.
Blinding	Salivary metabolome analyses were done by two of the co-authors who were blinded to the identity of the saliva. The investigators were also blinded to group allocation during RNA-seq data collection and/or analysis. It was not possible to do complete blinding for the in vitro and flow cytometry experiments, as the same research associate performed the cell culture and the staining. However, the person who did the flow-cytometry data analysis was blinded on the groups until the final analysis of all the replicate experiments at which point the research associate released the codes for the cell-culture groups.

Reporting for specific materials, systems and methods

We require information from authors about some types of materials, experimental systems and methods used in many studies. Here, indicate whether each material, system or method listed is relevant to your study. If you are not sure if a list item applies to your research, read the appropriate section before selecting a response.

Materials & experimental systems

n/a	Involved in the study	
☐	☒	Antibodies
☒	☐	Eukaryotic cell lines
☒	☐	Palaeontology and archaeology
☒	☐	Animals and other organisms
☒	☐	Clinical data
☒	☐	Dual use research of concern

Methods

n/a	Involved in the study	
☒	☐	ChIP-seq
☐	☒	Flow cytometry
☒	☐	MRI-based neuroimaging

Antibodies

Antibodies used

Antibodies, Source, Catalog number, Clone and Lot Dilution
CD28, Invitrogen, 16-0289-85, CD28.2, 2197855, 1:1000
CD4, Invitrogen, 56-00480-82, OKT4, 2114219, 1:200
CD45, Invitrogen, 61-0459-1, HI30 2135833, 1:500
HLA-DR, Invitrogen, 47-9956-42, LN3, 1950154, 1:100
IFN-γ, Invitrogen, 17-7319-82, 4S.B3, 2193918, 1:100
Ki-67, Invitrogen, 11-5698-82, SolA15, 2040334, 1:200
IL-10, Invitrogen, 46-7108-42, JES3-9D7, 4331074, 1:50
AREG, Invitrogen, 12-5370-42, AREG559 4351598, 1:50
Phospho-caspase 1 (Ser376), Invitrogen, PA5-38565, Polyclonal, UG2807841, 1:100
HIF-1a, BD Biosciences, 565924, 54/ HIF-1a, 0142782, 1:100
CD25, BD Biosciences, 563701, M-A251, 9081958, 1:200
CD8, BD Biosciences, 561453, RPA-T8, 0030878, 1:200
CCR6, BD Biosciences, 563923, 11A9, 7226510, 1:100
CD279 (PD-1), BD Biosciences, 564017, EH12.1, 0087488, 1:200
Bcl-2, BD Biosciences, 560637, Bcl-2/100, 9268283, 1:50
CD19 BD, Biosciences, 557835, SJ25C1, 6195788, 1:200
CD38 BD, Biosciences, 563964, HIT2, 7089805, 1:200
CD3 BD, Biosciences, 740073, HIT3a, 1049330, 1:1000
IL-1R1 (hIL1R-M1), BD Biosciences, 551388, CD121a (IL-1R1), 9297644
ODC-1, Novus Biologicals, NBP2-34700PCP, ODC1/485, 4953-1PABx210629, 1:50
IL-17A, eBioscience, 47-7179-42, eBio64, 1952434, 1:50
FOXP3, eBioscience, 14-4777-82, 236A/E7, 4341581, 1:50
CXCR5, eBioscience, 48-9185-43, MU5UBEE, 4275264, 1:200
NLRP3, R and D systems, IC7578P, 768319, ACZF0221051, 1:200
EIF5A, Thermo fisher, MA1-179, Recombinant expressed in E. coli, RJ240038, 1:100
IL-1β Thermo fisher, 12-7018-82, CRM56, 2265337, 1:50
Hypusine, Millipore Sigma ABS1064 Polyclonal 3748850, 1:50
ROR-gt, Life technologies, 46-6988-82, AFKJS-9, 2140726, 1:100
LC3B, R and D systems, Human LC3B Alexa Fluor® 647-conjugated Antibody IC9390R; 1:50
Secondary donkey anti-mouse IgG-BV421, Jackson ImmunoResearch, 715-675-150, 145349, 1:500
Anti-rabbit-PE, Caltag Laboratories, L43004, 1601, 1:800

Validation

All antibodies were commercially available, validated and were used in previous studies, as per the references available in manufacturer's websites. For the BD- Biosciences flow cytometry antibodies, the company website says "The production process underwent stringent testing and validation to assure that it generates a high-quality conjugate with consistent performance and specific binding activity". The invitrogen/Thermofisher website says: "To help ensure superior antibody results, we've expanded our specificity testing methodology using a 2-part approach for advanced verification".

We also titrated the antibodies using unstained or Isotype controls prior to use.

Flow Cytometry

Plots

Confirm that:

☒ The axis labels state the marker and fluorochrome used (e.g. CD4-FITC).

☒ The axis scales are clearly visible. Include numbers along axes only for bottom left plot of group (a 'group' is an analysis of identical markers).

☒ All plots are contour plots with outliers or pseudocolor plots.

☒ A numerical value for number of cells or percentage (with statistics) is provided.

Methodology

Sample preparation	For single-cell flow cytometry staining, cells isolated and processed ex vivo from tissues or lymphoid organs as well as cultured cells were washed in PBS or PBS/BSA, and blocked by Fc receptor blocking, before surface staining using the antibodies. For Foxp3 and other intracellular marker staining, the cells were fixed with Foxp3 fix-perm set (eBioSciences/ Thermofisher) after surface staining. Live-Dead viability staining was used to remove dead cells in the analyses. Appropriate un-stain, isotype, secondary antibody, single stain and FMO controls were used and representative data are shown in supplementary figures. Before intracellular cytokine staining, cultures were re-stimulated with PMA (50 ng/ml) and Ionomycin (500 ng/ml) for 4 hours, with brefeldin-A (10 μg/ml) added in last 2 hours. For phospho staining, the cells were washed, fixed and were stained with Phosflow staining kit from BD Biosciences using manufacturer's protocol.
Instrument	BD Fortessa
Software	Flowjo versions 9.8, 9.9.6, 10.5.3 and 10.7.1
Cell population abundance	CD4+ T ells were more abundant in tonsils than in the oral tissues.
Gating strategy	Gating strategy: Preliminary FSC/SSC gates for the starting leukocyte cell population, and subsequent gating to include singlets, and CD3+ T cells were used. Boundaries of the "positive" gates were assigned based on the unstained controls, PBMC/d0 negative controls, and FMO controls. We have shown data exemplifying the gating strategies and the controls where appropriate in the supplementary Information.

☒ Tick this box to confirm that a figure exemplifying the gating strategy is provided in the Supplementary Information.
